# Hydroxylation of Aryl Sulfonium Salts for Phenol Synthesis under Mild Reaction Conditions

**DOI:** 10.3390/molecules29040831

**Published:** 2024-02-13

**Authors:** Xuan-Bo Hu, Qian-Qian Fu, Xue-Ying Huang, Xue-Qiang Chu, Zhi-Liang Shen, Chengping Miao, Weiyi Chen

**Affiliations:** 1 Technical Institute of Fluorochemistry (TIF), School of Chemistry and Molecular Engineering, Nanjing Tech University, Nanjing 211816, China; hxb1314521@njtech.edu.cn (X.-B.H.); fu200122@njtech.edu.cn (Q.-Q.F.); huangxueying@njtech.edu.cn (X.-Y.H.); xueqiangchu@njtech.edu.cn (X.-Q.C.); 2 College of Biological, Chemical Science and Engineering, Jiaxing University, 118 Jiahang Road, Jiaxing 314001, China; 3 Soochow College, Soochow University, Suzhou 215006, China

**Keywords:** hydroxylation, phenol synthesis, aryl sulfonium salts, acetohydroxamic acid, oxime

## Abstract

Hydroxylation of aryl sulfonium salts could be realized by utilizing acetohydroxamic acid and oxime as hydroxylative agents in the presence of cesium carbonate as a base, leading to a variety of structurally diverse hydroxylated arenes in 47–95% yields. In addition, the reaction exhibited broad functionality tolerance, and a range of important functional groups (e.g., cyano, nitro, sulfonyl, formyl, keto, and ester) could be well amenable to the mild reaction conditions.

## 1. Introduction

**Scheme 1:**
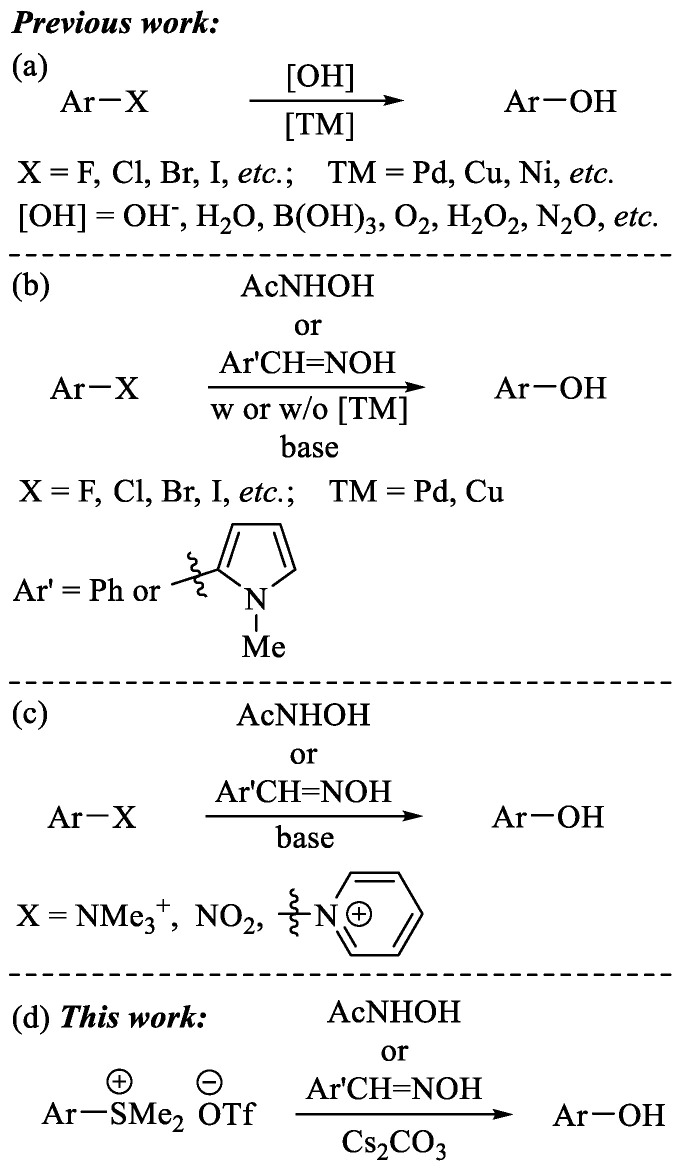
Hydroxylation of aryl halides and their counterparts. (**a**) Hydroxylation of aryl halides by using various hydroxide sources [11,12,13,14,15,16,17,18,19,20,21,22,23,24,25,26,27,28,29,30,31,32,33]; (**b**) Hydroxylation of aryl halides by using oxime and acetohydroxamic acid [34,35,36,37,38,39]; (**c**) Hydroxylation of other aryl electrophiles by using oxime and hydroxamic acid [48,49,50]; (**d**) Hydroxylation of aryl sulfonium salts by using oxime and hydroxamic acid (this work).

Phenols and their derivatives are not only important building blocks in modern organic synthesis, but also structural units which are ubiquitously present in natural products, biologically and pharmaceutically active molecules, and functional materials [1]. As a consequence, the development of effective methodologies for the preparation of phenols has attracted considerable attention from the synthetic community. In addition to the classic methods developed in order to access phenols by virtue of Sandmeyer reactions and direct arene oxidation [2,3,4,5,6,7,8,9,10], the transition-metal-catalyzed hydroxylation of aryl halides has also been proven to be one of the most efficient methods by which to synthesize phenol [11,12,13,14,15,16,17]. A range of hydroxide sources and their surrogates, such as alkali metal hydroxide (MOH) [18,19,20,21], water [22,23,24,25,26,27], boric acid [28], molecular oxygen [29,30], silanol [31], hydrogen peroxide [31], and nitrous oxide [32,33], have been revealed to be effective for the hydroxylation of aryl halides and its variants (Scheme 1a). In 2016 and 2017, the group of Fier and Maloney originally disclosed that benzaldoxime [34,35,36,37] and acetohydroxamic acid [38] could also function as effective hydroxylating agents for the conversion of aryl halides to phenols (Scheme 1b). In 2021, James et al. found that electron-rich pyrrole-based oxime is more efficient than benzaldoxime for the hydroxylation of aryl halides [39].

Recently, the development of alternative electrophiles [40,41,42,43,44,45,46,47] as substitutes for conventional aryl halides in hydroxylative reactions have aroused considerable attention in the field of synthetic organic chemistry (Scheme 1c) [48,49,50]. For instance, Cornella et al. have described how pyridinium salts generated in situ from the reaction of aminoheterocycles with pyrylium tetrafluoroborate salts could be converted into their hydroxylated analogue by utilizing acetohydroxamic acid as a hydroxyl source [48]. James et al. have demonstrated that nitroarenes could also be effectively transformed into phenols via a denitrative functionalization protocol that employs their previously developed pyrrole-based oxime as hydroxylating agent under transition-metal-free conditions [49]. Cheng and Ye have reported that aryl ammonium salts could also efficiently undergo hydroxylation by using benzaldoxime and acetohydroxamic acid as hydroxide surrogates [50]. In recent decades, readily accessible and shelf-stable organosulfonium salts have also been proven to be versatile electrophiles for undergoing a broad range of organic transformations [51,52,53,54,55,56,57,58,59,60,61,62,63,64,65,66,67,68,69,70,71,72,73]. Although the hydroxylation of aryl thianthrenium salts have been accomplished by using water as hydroxide source, the reaction should be conducted under photoredox conditions in the presence of Ir and Cu catalysts [74]. In the continuation of our efforts to develop efficient organic transformations with the use of alternative electrophiles [75,76,77,78,79,80,81,82,83,84] under mild reaction conditions, herein we report a hydroxylation of aryl sulfonium salts by using acetohydroxamic acid and oxime as hydroxylative agents, which enabled the efficient assembly of hydroxylated arenes in modest-to-good yields with good functional group compatibility (Scheme 1d).

## 2. Results

Initially, we sought to optimize the reaction conditions for the hydroxylation of (4-cyanophenyl)dimethylsulfonium-trifluoromethanesulfonate (**1a**) by using *N*-hydroxyacetamide (**2a**) as a hydroxylating agent [38] in the co-existence of various bases and solvents. Among the different organic bases and inorganic bases surveyed and outlined in Table 1 (entries 1–11), Cs_2_CO_3_ emerged as the base of choice (entry 8), leading to the corresponding phenol **3a** in 66% NMR yield when the reaction was carried out in DMSO (1 mL) at 80 °C for 18 h. Subsequent screening of other reaction media, including DMF, 1,4-dioxane, NMP, MeCN, toluene, THF, and H_2_O (entries 12–18), did not further enhance the reaction performance. Conducting the reaction at different temperatures (60 °C and 100 °C, entries 19–20) also made no significant difference. Gratifyingly, the NMR yield of product **3a** could be further improved to 81% by using 2 mL DMSO as solvent (entry 21). In addition, evaluation of other reaction parameters, including the amount of Cs_2_CO_3_ (3 equiv. or 7 equiv., entries 22–23), reaction time (12 h or 24 h, entries 24–25), equivalents of *N*-hydroxyacetamide **2a** (2 equiv. or 4 equiv., entries 26–27), and the amount of DMSO (4 mL, entry 28), were performed. However, in most cases, variation of the reaction conditions either led to decreased product yield or produced a comparable result to that of entry 21.

Under the above established reaction conditions, we investigated an array of aryl sulfonium salts for this hydroxylation reaction by utilizing acetohydroxamic acid **2a** as the hydroxylating agent. As summarized in Table 2, aryl sulfonium salts **1a–k**, containing an electron-withdrawing group in the aryl ring, efficiently took part in the hydroxylation to give the corresponding phenols at moderate-to-high yields. More significantly, important functional groups, including cyano, nitro, sulfonyl, formyl, keto, and ester, could be well compatible with the established conditions, which could be retained for downstream derivatization. However, when (4-chlorophenyl)dimethylsulfonium triflate containing a chloro atom in the phenyl ring was used as a substrate, no desired hydroxylative product was obtained, presumably because of its relatively low reactivity as compared with aryl sulfonium salt bearing an electron-withdrawing group in the phenyl ring. In addition, and analogous to a previous report of Fier and Maloney in which the hydroxylation only worked with an electron-poor aryl halide [38], the present reaction also could not be applied to less reactive aryl sulfonium salts derived from an electron-rich aryl ring which bears an electron-donating substituent.

In addition to acetohydroxamic acid **2a**, we also investigated the hydroxylation of aryl sulfonium salt **1a** by employing benzaldoxime [34,35,36,37,39] (1.3 equiv.) as a hydroxylative reagent. In the beginning, we also optimized the reaction conditions. As outlined in Table 3, the reaction of aryl sulfonium salt **1a** with benzaldehyde oxime (**2b**) proceeded smoothly in the presence of Cs_2_CO_3_ in DMSO at 80 °C for 4 h to give the hydroxylated product **3a** in 58% NMR yield (entry 1). Ensuing screening of reaction solvent (entries 2–7) revealed that DMF served as the more appropriate solvent of the reaction, which slightly improved the reaction efficiency and gave rise to the corresponding product **3a** in 67% NMR yield (entry 7). Next, a variety of bases were also examined in the hydroxylating reaction (entries 8–19). However, Cs_2_CO_3_ was still the optimal base for the transformation. Pleasingly, by increasing the amount of **2b** to 1.5 equivalents, NMR yield of the product could be increased to 74% (entry 20).

Apart from benzaldehyde oxime (**2b**), a variety of oximes **2** were also evaluated as hydroxide surrogates (Table 4). Of the various oximes **2b**–**g** studied, oxime **2g**, derived from pyrrole [39], was found to be the most suitable hydroxylating reagent for the reaction, affording the expected product **3a** in 89% NMR yield.

With the establishment of the optimized reaction conditions, substrate scope of aryl sulfonium salts **1** was investigated with the use of oxime **2g** as the hydroxylating agent. As listed in Table 5, a variety of aryl sulfonium salts **1a**–**k** possessing an electron-poor phenyl ring could be amenable to the reaction, leading to the corresponding hydroxylated arenes in 47–95% yields. Analogously, the reactions proceeded with tolerances to a plethora of important functionalities, such as CN, NO_2_, SO_2_Me, CHO, COR, and COOR. Additionally, aryl sulfonium salts containing electron-donating group in the aryl ring were proven to be inappropriate for the current hydroxylation reaction, which is similar to reports in the literature [38,39,49,50].

**Scheme 2:**
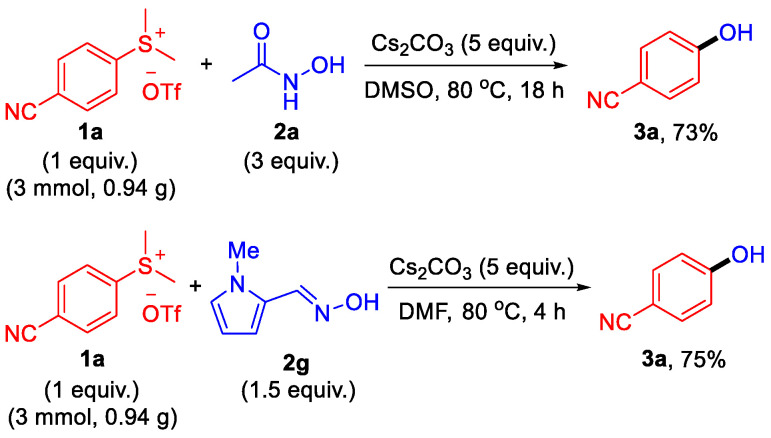
Scale-up synthesis.

Finally, the scalability of the reactions was also investigated. As illustrated in Scheme 2, 3 mmol scale reaction of aryl sulfonium salt **1a** with both **2a** and **2g** worked equally well under the optimized reaction conditions, producing the anticipated product **3a** in 73% and 75% yields, respectively.

**Figure 1:**
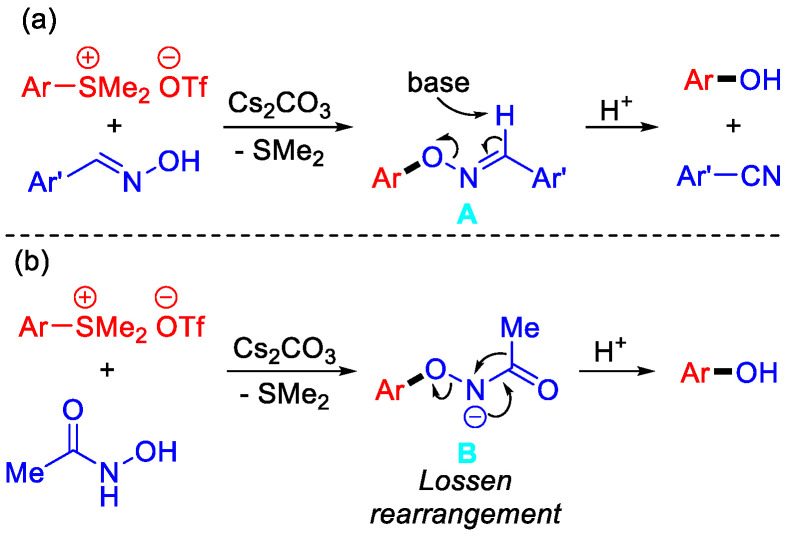
Tentatively proposed mechanisms. (**a**) When using aldehyde oxime as hydroxylative source; (**b**) When using acetohydroxamic acid as hydroxylative source.

Based on previous reports [34,38,50], possible mechanisms for these two hydroxylative reactions have been tentatively proposed. As shown in Figure 1a, for the reaction of aryl sulfonium salt with aldehyde oxime, the reaction possibly proceeds via the nucleophilic substitution of the aldehyde oxime with the sulfonium salt under the action of base to give intermediate **A**, accompanied by the generation of dimethyl sulfide as a byproduct. Next, a base-mediated deprotonation of intermediate **A**, followed by fragmentation and subsequent protonation, affords the corresponding phenol as the final product and aryl nitrile as a byproduct. With regard to hydroxylation employing acetohydroxamic acid as hydroxide source (Figure 1b), the reaction presumably occurs through a similar base-facilitated nucleophilic substitution of acetohydroxamic acid with aryl sulfonium salt to produce intermediate **B**, along with the formation of Me_2_S as a byproduct. Subsequently, a Lossen rearrangement takes place to yield the desired phenol after acidific workup.

## 3. Materials and Methods

### 3.1. General Information

Unless otherwise specified, the reagents were purchased from commercial suppliers and used without further purification. All reactions were conducted under N_2_ atmosphere using undistilled solvent. Analytical thin layer chromatography (TLC) was performed using silica gel plate (0.2 mm thickness). Subsequent to elution, plates were visualized using UV radiation (254 nm). Flash chromatography was performed using Merck silica gel (200–300 mesh) for column chromatography with freshly distilled solvents. IR spectra were recorded on an FT-IR spectrophotometer using KBr optics. ^1^H and ^13^C NMR spectra were recorded in CDCl_3_ and DMSO-d^6^ on Bruker Avance or Jeol 400 MHz spectrometers. The chemical shifts (δ) are reported in ppm and coupling constants (*J*) in Hz. NMR splitting patterns are designated as singlet (s), doublet (d), triplet (t), quartet (q), multiplet (m), doublet of doublets (dd), doublet of triplets (dt), doublet of quartets (dq), triplet of doublets (td), triplet of triplets (tt), quartet of doublets (qd), doublet of doublet of doublets (ddd), etc. Tetramethylsilane (TMS) served as internal standard for ^1^H and ^13^C NMR analysis. High resolution mass spectra (HRMS) were obtained via a Waters Q-TOF Premier Spectrometer (ESI source).

### 3.2. Experimental

#### 3.2.1. General Procedure for the Synthesis of Aryl Sulfonium Salts **1a**–**k**

A 50 mL single-neck round-bottom flask was sequentially charged with aryl thioether (20 mmol, 1.0 equiv.), DCE (20 mL), and MeOTf (3938.4 mg, 24 mmol, 1.2 equiv.). The reaction mixture was vigorously stirred at room temperature for 12 h. Then, solvent was evaporated and the residual was purified through silica gel column chromatography using petroleum ether and EtOAc as eluent to afford the analytically pure product of aryl sulfonium salts **1a**–**k**. Spectral data of these compounds are in accordance with those previously documented [62,63,64,65].

#### 3.2.2. General Procedure for the Synthesis of Oximes **2b**–**g**

A 250 mL round-bottom flask was sequentially charged with aryl aldehyde (20.0 mmol, 1 equiv.), MeOH (100 mL), Na_2_CO_3_ (2.54 g, 24 mmol, 1.2 equiv.), and NH_2_OH∙HCl (1.67 g, 24 mmol, 1.2 equiv.). The reaction mixture was then heated to reflux and stirred for 2 h. The reaction was allowed to cool to room temperature and MeOH was removed under reduced pressure. The residue was dissolved in EtOAc (50 mL) and H_2_O (50 mL). The organic phase was separated and the aqueous phase was extracted with EtOAc (50 mL × 2). The organic extracts were combined, washed with brine (50 mL), and dried over Na_2_SO_4_. The extracts were concentrated under reduced pressure to afford the crude product, which was purified via silica gel column chromatography (using EtOAc/petroleum ether + 3% Et_3_N as eluents) to afford the analytically pure oximes **2b**–**g**. Spectral data of the products are in accordance with those previously documented [39].

#### 3.2.3. General Procedure for the Reaction of Aryl Sulfonium Salt with Acetohydroxamic Acid

To an oven-dried Schlenk tube equipped with a magnetic stir bar was sequentially added aryl sulfonium salt **1** (0.5 mmol, 1 equiv.), acetohydroxamic acid **2a** (112.6 mg, 1.5 mmol, 3 equiv.), and Cs_2_CO_3_ (814.6 mg, 2.5 mmol, 5 equiv.). Then, dry DMSO (2 mL) was added into the tube by syringe. The reaction mixture was stirred at 80 °C for 18 h before quenching with aqueous hydrochloric acid (6 mmol, 6 mL, 1 M in water) and extracting with EtOAc (20 mL × 3). The organic layers were combined, washed with brine, and dried over Na_2_SO_4_. The extracts were concentrated under reduced pressure to afford the crude product, which was further purified via silica gel column chromatography (using EtOAc/petroleum ether as eluents) to yield the analytically pure product **3**.

#### 3.2.4. General Procedure for the Reaction of Aryl Sulfonium Salt with Oxime

To an oven-dried Schlenk tube equipped with a magnetic stir bar was sequentially added aryl sulfonium salt **1** (0.5 mmol, 1 equiv.), oxime **2g** (93.1 mg, 0.75 mmol, 1.5 equiv.), and Cs_2_CO_3_ (814.6 mg, 2.5 mmol, 5 equiv.). Then dry DMF (2 mL) was added into the tube by syringe. The reaction mixture was stirred at 80 °C for 4 h before quenching with aqueous hydrochloric acid (6 mmol, 6 mL, 1 M in water) and extracting with EtOAc (20 mL × 3). The organic layers were combined, washed with brine, and dried over Na_2_SO_4_. The extracts were concentrated under reduced pressure to afford the crude product, which was further purified through silica gel column chromatography (using EtOAc/petroleum ether as eluents) to yield the analytically pure product **3**.

#### 3.2.5. Scale-Up Reaction of Sulfonium Salt **1a** with Acetohydroxamic Acid **2a**

To an oven-dried Schlenk tube equipped with a magnetic stir bar was sequentially added aryl sulfonium salt **1a** (940.0 mg, 3 mmol, 1 equiv.), acetohydroxamic acid **2a** (1013.4 mg, 9 mmol, 3 equiv.), and Cs_2_CO_3_ (7331.4 mg, 15 mmol, 5 equiv.). Then dry DMSO (12 mL) was added into the tube by syringe. The reaction mixture was stirred at 80 °C for 18 h before quenching with aqueous hydrochloric acid (36 mmol, 36 mL, 1 M in water) and extracting with EtOAc (80 mL × 3). The organic layers were combined, washed with brine, and dried over Na_2_SO_4_. The extracts were concentrated under reduced pressure to afford the crude product, which was further purified through silica gel column chromatography (using EtOAc/petroleum ether as eluents) to yield the analytically pure product **3a** in 73% yield (262.9 mg).

#### 3.2.6. Scale-Up Reaction of Sulfonium Salt **1a** with Oxime **2g**

To an oven-dried Schlenk tube equipped with a magnetic stir bar was sequentially added aryl sulfonium salt **1a** (940.0 mg, 3 mmol, 1 equiv.), oxime **2g** (419.0 mg, 4.5 mmol, 1.5 equiv.), and Cs_2_CO_3_ (7331.4 mg, 15 mmol, 5 equiv.). Then, dry DMSO (12 mL) was added into the tube by syringe. The reaction mixture was stirred at 80 °C for 4 h before quenching with aqueous hydrochloric acid (36 mmol, 36 mL, 1 M in water) and extracting with EtOAc (80 mL × 3). The organic layers were combined, washed with brine, and dried over Na_2_SO_4_. The extracts were concentrated under reduced pressure to afford the crude product, which was further purified through silica gel column chromatography (using EtOAc/petroleum ether as eluents) to yield the analytically pure product **3a** in 75% yield (270.1 mg).

*4-Hydroxybenzonitrile* (**3a**). Purification of this product was performed via silica gel column chromatography employing petroleum ether and ethyl acetate as eluant (petroleum ether/EtOAc = 15:1) with a yield = 86%, 51.4 mg using **2a** as hydroxylating agent and a yield = 87%, 51.8 mg using **2g** as hydroxylating agent. White solid. ^1^H NMR (400 MHz, CDCl_3_): δ 7.57–7.51 (m, 2H), 7.18 (brs, 1H), 6.97–6.90 (m, 2H) ppm. ^13^C NMR (100 MHz, CDCl_3_): δ 160.4, 134.3, 119.3, 116.5, 102.6 ppm. IR (KBr, neat): *ν* = 3292, 2927, 2856, 1610, 1586, 1508, 1286, 1166, 835, 700, 458 cm^−1^. HRMS (ESI, *m*/*z*): calculated for C_7_H_6_NO [M + H]^+^ 120.0444, found: 120.0442. Spectral data of the product are in accordance with previously documented data [85].

*4-Hydroxy-2-methylbenzonitrile* (**3b**). Purification of this product was performed via silica gel column chromatography employing petroleum ether and ethyl acetate as eluant (petroleum ether/EtOAc = 20:1) with a yield = 49%, 32.9 mg using **2a** as hydroxylating agent and a yield = 59%, 39.5 mg using **2g** as hydroxylating agent. Yellow solid. ^1^H NMR (400 MHz, CDCl_3_): δ 7.48 (d, *J* = 8.4 Hz, 1H), 6.78 (d, *J* = 2.5 Hz, 1H), 6.74 (dd, *J* = 8.4, 2.5 Hz, 1H), 6.47 (brs, 1H), 2.48 (s, 3H) ppm. ^13^C NMR (100 MHz, CDCl_3_): δ 159.9, 144.5, 134.5, 118.6, 117.2, 113.8, 103.8, 20.5 ppm. IR (KBr, neat): *ν* = 3300, 2921, 2225, 1616, 1576, 1498, 1303, 1231, 1163, 1098 cm^−1^. HRMS (ESI, *m*/*z*): calculated for C_8_H_8_KNO [M+K]^+^ 172.0159, found: 172.0170. Spectral data of the product are in accordance with previously documented data [24].

*4-Nitrophenol* (**3c**). Purification of this product was performed via silica gel column chromatography employing petroleum ether and ethyl acetate as eluant (petroleum ether/EtOAc = 20:1) with a yield = 95%, 66.3 mg using **2a** as hydroxylating agent and a yield = 95%, 66.1 mg using **2g** as hydroxylating agent. Yellow solid. ^1^H NMR (400 MHz, CDCl_3_): δ 8.21–8.13 (m, 2H), 6.98–6.91 (m, 2H), 6.64 (brs, 1H) ppm. ^13^C NMR (100 MHz, CDCl_3_): δ 161.7, 141.4, 126.3, 115.8 ppm. IR (KBr, neat): *ν* = 3337, 2962, 2360, 2342, 1592, 1498, 1339, 1289, 1262, 1112 cm^−1^. HRMS (ESI, *m*/*z*): calculated for C_6_H_6_NO_3_ [M + H]^+^ 140.0342, found: 140.0353. Spectral data of the product are in accordance with previously documented data [85].

*3-Methyl-4-nitrophenol* (**3d**). Purification of this product was performed via silica gel column chromatography employing petroleum ether and ethyl acetate as eluant (petroleum ether/EtOAc = 20:1) with a yield = 79%, 60.5 mg using **2a** as hydroxylating agent and a yield = 55%, 42.4 mg using **2g** as hydroxylating agent. White solid. ^1^H NMR (400 MHz, DMSO-*d*_6_): δ 10.79 (brs, 1H), 8.00–7.95 (m, 1H), 6.79–6.74 (m, 2H), 2.49 (s, 3H) ppm. ^13^C NMR (100 MHz, DMSO-*d*_6_): δ 162.7, 140.9, 137.3, 128.3, 119.2, 114.3, 21.5 ppm. IR (KBr, neat): *ν* = 3301, 3090, 2929, 1589, 1507, 1477, 1458, 1317, 1260, 1077 cm^−1^. HRMS (ESI, *m*/*z*): calculated for C_7_H_8_NO_3_ [M + H]^+^ 154.0499, found: 154.0498. Spectral data of the product are in accordance with previously documented data [86].

*4-(Methylsulfonyl)phenol* (**3e**). Purification of this product was performed via silica gel column chromatography employing petroleum ether and ethyl acetate as eluant (petroleum ether/EtOAc = 5:1) with a yield = 81%, 70.1 mg using **2a** as hydroxylating agent and a yield = 79%, 68.3 mg using **2g** as hydroxylating agent. Yellow solid. ^1^H NMR (400 MHz, CDCl_3_): δ 7.78–7.65 (m, 3H), 7.00–6.92 (m, 2H), 3.06 (s, 3H) ppm. ^13^C NMR (100 MHz, CDCl_3_): δ 161.4, 130.6, 129.6, 116.2, 44.9 ppm. IR (KBr, neat): *ν* = 3375, 3017, 2918, 1588, 1502, 1447, 1302, 1283, 1144, 1090 cm^−1^. HRMS (ESI, *m*/*z*): calculated for C_7_H_9_O_3_S [M + H]^+^ 173.0267, found: 173.0266. Spectral data of the product are in accordance with previously documented data [24].

*4-Hydroxybenzaldehyde* (**3f**). Purification of this product was performed via silica gel column chromatography employing petroleum ether and ethyl acetate as eluant (petroleum ether/EtOAc = 20:1) with a yield = 61%, 37.3 mg using **2a** as hydroxylating agent and a yield = 52%, 31.8 mg using **2g** as hydroxylating agent. White solid. ^1^H NMR (400 MHz, CDCl_3_): δ 9.84 (s, 1H), 7.86–7.78 (m, 2H), 7.38 (brs, 1H), 7.04–6.97 (m, 2H) ppm. ^13^C NMR (100 MHz, CDCl_3_): δ 191.9, 162.4, 132.8, 129.3, 116.1 ppm. IR (KBr, neat): *ν* = 3167, 2878, 1668, 1599, 1453, 1316, 1287, 1218, 1161, 834 cm^−1^. HRMS (ESI, *m*/*z*): calculated for C_7_H_7_O_2_ [M + H]^+^ 123.0441, found: 123.0453. Spectral data of the product are in accordance with previously documented data [87].

*(4-Hydroxyphenyl)(phenyl)methanone* (**3g**). Purification of this product was performed via silica gel column chromatography employing petroleum ether and ethyl acetate as eluant (petroleum ether/EtOAc = 20:1) with a yield = 56%, 55.9 mg using **2a** as hydroxylating agent) and a yield = 53%, 52.8 mg using **2g** as hydroxylating agent. Yellow solid. ^1^H NMR (400 MHz, CDCl_3_): δ 7.82–7.73 (m, 4H), 7.65 (brs, 1H), 7.61–7.55 (m, 1H), 7.52–7.44 (m, 2H), 6.99–6.91 (m, 2H) ppm. ^13^C NMR (100 MHz, CDCl_3_): δ 196.8, 160.8, 138.0, 133.1, 132.2, 129.8, 129.5, 128.3, 115.4 ppm. IR (KBr, neat): *ν* = 3150, 2346, 1634, 1602, 1559, 1507, 1313, 1288, 1232, 1148 cm^−1^. HRMS (ESI, *m*/*z*): calculated for C_13_H_11_O_2_ [M + H]^+^ 199.0754, found: 199.0765. Spectral data of the product are in accordance with previously documented data [24].

*2-Hydroxyanthracene-9,10-dione* (**3h**). Purification of this product was performed via silica gel column chromatography employing petroleum ether and ethyl acetate as eluant (petroleum ether/EtOAc = 20:1) with a yield = 73%, 81.8 mg using **2a** as hydroxylating agent and a yield = 68%, 76.5 mg using **2g** as hydroxylating agent. Yellow solid. ^1^H NMR (400 MHz, CDCl_3_): δ 8.17–8.14 (m, 2H), 8.08 (d, *J* = 8.5 Hz, 1H), 7.93–7.85 (m, 2H), 7.49 (d, *J* = 2.5 Hz, 1H), 7.24 (dd, *J* = 8.5, 2.6 Hz, 1H) ppm. ^13^C NMR (100 MHz, CDCl_3_): δ 183.0, 181.5, 163.6, 135.6, 134.9, 134.4, 133.6, 133.5, 130.3, 127.03, 127.01, 125.6, 122.0, 112.7 ppm. IR (KBr, neat): *ν* = 3357, 2960, 2255, 1671, 1581, 1450, 1342, 1304, 1022, 997 cm^−1^. HRMS (ESI, *m*/*z*): calculated for C_14_H_9_O_3_ [M + H]^+^ 225.0546, found: 225.0551. Spectral data of the product are in accordance with previously documented data [88].

*1-(4-Hydroxyphenyl)ethan-1-one* (**3i**). Purification of this product was performed via silica gel column chromatography employing petroleum ether and ethyl acetate as eluant (petroleum ether/EtOAc = 20:1) with a yield = 51%, 34.7 mg using **2a** as hydroxylating agent and a yield = 60%, 41.0 mg using **2g** as hydroxylating agent. White solid. ^1^H NMR (400 MHz, CDCl_3_): δ 8.15 (brs, 1H), 7.95–7.88 (m, 2H), 6.97–6.92 (m, 2H), 2.58 (s, 3H) ppm. ^13^C NMR (100 MHz, CDCl_3_): δ 199.0, 161.7, 131.3, 129.3, 115.6, 26.3 ppm. IR (KBr, neat): *ν* = 3306, 2995, 2926, 1663, 1603, 1577, 1512, 1357, 1279, 1219, 1166 cm^−1^. HRMS (ESI, *m*/*z*): calculated for C_8_H_9_O_2_ [M + H]^+^ 137.0597, found: 137.0594. Spectral data of the product are in accordance with previously documented data [85].

*Methyl 4-hydroxybenzoate* (**3j**). Purification of this product was performed via silica gel column chromatography employing petroleum ether and ethyl acetate as eluant (petroleum ether/EtOAc = 20:1) with a yield = 54%, 41.1 mg using **2a** as hydroxylating agent and a yield = 47%, 35.8 mg using **2g** as hydroxylating agent. White solid. ^1^H NMR (400 MHz, CDCl_3_): δ 7.97–7.91 (m, 2H), 7.15 (brs, 1H), 6.93–6.87 (m, 2H), 3.90 (s, 3H) ppm. ^13^C NMR (100 MHz, CDCl_3_): δ 167.8, 160.6, 132.0, 121.9, 115.3, 52.2 ppm. IR (KBr, neat): *ν* = 3307, 2962, 1683, 1607, 1588, 1515, 1435, 1278, 1163, 1106 cm^−1^. HRMS (ESI, *m*/*z*): calculated for C_8_H_9_O_3_ [M + H]^+^ 153.0546, found: 153.0549. Spectral data of the product are in accordance with previously documented data [85].

*5-Hydroxyisobenzofuran-1(3H)-one* (**3k**)**.** Purification of this product was performed via silica gel column chromatography employing petroleum ether and ethyl acetate as eluant (petroleum ether/EtOAc = 20:1) with a yield = 70%, 52.8 mg using **2a** as hydroxylating agent and a yield = 60%, 45.1 mg using **2g** as hydroxylating agent. Yellow solid. ^1^H NMR (400 MHz, DMSO-*d*_6_): δ 10.67 (brs, 1H), 7.70–7.60 (m, 1H), 6.98–6.89 (m, 2H), 5.26 (s, 2H) ppm. ^13^C NMR (100 MHz, DMSO-*d*_6_): δ 171.0, 163.6, 150.8, 127.2, 117.6, 116.1, 108.9, 69.6 ppm. IR (KBr, neat): *ν* = 3270, 2962, 2925, 1718, 1603, 1467, 1434, 1346, 1273, 1097 cm^−1^. HRMS (ESI, *m*/*z*): calculated for C_8_H_7_O_3_ [M + H]^+^ 151.0390, found: 151.0399. Spectral data of the product are in accordance with previously documented data [32].

## 4. Conclusions

In conclusion, the hydroxylation of aryl sulfonium salts by utilizing acetohydroxamic acid and oxime as hydroxylating reagents was developed. The reactions proceeded effectively with the aid of cesium carbonate to afford a series of hydroxylated arenes in moderate-to-high yields with broad functional group compatibility. In addition, the hydroxylation could be subjected to scale-up synthesis, leading to the desired phenol in a good yield.

## Figures and Tables

**Table 1:** Optimization of reaction conditions ^a^.

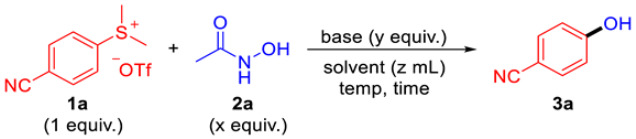						
Entry	2a (x equiv.)	Base (y equiv.)	Solvent (z mL)	Temp. (°C)	Time (h)	Yield (%) ^b^
1	3 equiv.	DBU (5 equiv.)	DMSO (1 mL)	80 °C	18 h	9
2	3 equiv.	DABCO (5 equiv.)	DMSO (1 mL)	80 °C	18 h	<5
3	3 equiv.	CsF (5 equiv.)	DMSO (1 mL)	80 °C	18 h	31
4	3 equiv.	NaOAc (5 equiv.)	DMSO (1 mL)	80 °C	18 h	<5
5	3 equiv.	K_3_PO_4_ (5 equiv.)	DMSO (1 mL)	80 °C	18 h	27
6	3 equiv.	Na_2_CO_3_ (5 equiv.)	DMSO (1 mL)	80 °C	18 h	10
7	3 equiv.	K_2_CO_3_ (5 equiv.)	DMSO (1 mL)	80 °C	18 h	52
8	3 equiv.	Cs_2_CO_3_ (5 equiv.)	DMSO (1 mL)	80 °C	18 h	66
9	3 equiv.	KOH (5 equiv.)	DMSO (1 mL)	80 °C	18 h	40
10	3 equiv.	*^t^*BuOK (5 equiv.)	DMSO (1 mL)	80 °C	18 h	6
11	3 equiv.	LDA (5 equiv.)	DMSO (1 mL)	80 °C	18 h	11
12	3 equiv.	Cs_2_CO_3_ (5 equiv.)	DMF (1 mL)	80 °C	18 h	53
13	3 equiv.	Cs_2_CO_3_ (5 equiv.)	1,4-dioxane (1 mL)	80 °C	18 h	11
14	3 equiv.	Cs_2_CO_3_ (5 equiv.)	NMP (1 mL)	80 °C	18 h	56
15	3 equiv.	Cs_2_CO_3_ (5 equiv.)	MeCN (1 mL)	80 °C	18 h	24
16	3 equiv.	Cs_2_CO_3_ (5 equiv.)	toluene (1 mL)	80 °C	18 h	6
17	3 equiv.	Cs_2_CO_3_ (5 equiv.)	THF (1 mL)	80 °C	18 h	5
18	3 equiv.	Cs_2_CO_3_ (5 equiv.)	H_2_O (1 mL)	80 °C	18 h	<5
19	3 equiv.	Cs_2_CO_3_ (5 equiv.)	DMSO (1 mL)	60 °C	18 h	66
20	3 equiv.	Cs_2_CO_3_ (5 equiv.)	DMSO (1 mL)	100 °C	18 h	69
21	3 equiv.	Cs_2_CO_3_ (5 equiv.)	DMSO (2 mL)	80 °C	18 h	81 (86) ^c^
22	3 equiv.	Cs_2_CO_3_ (3 equiv.)	DMSO (2 mL)	80 °C	18 h	78
23	3 equiv.	Cs_2_CO_3_ (7 equiv.)	DMSO (2 mL)	80 °C	18 h	81
24	3 equiv.	Cs_2_CO_3_ (5 equiv.)	DMSO (2 mL)	80 °C	12 h	80
25	3 equiv.	Cs_2_CO_3_ (5 equiv.)	DMSO (2 mL)	80 °C	24 h	82
26	2 equiv.	Cs_2_CO_3_ (5 equiv.)	DMSO (2 mL)	80 °C	18 h	69
27	4 equiv.	Cs_2_CO_3_ (5 equiv.)	DMSO (2 mL)	80 °C	18 h	79
28	3 equiv.	Cs_2_CO_3_ (5 equiv.)	DMSO (4 mL)	80 °C	18 h	86

^a^ Unless otherwise specified, the reactions were conducted at 80 °C for 18 h by employing **1a** (0.5 mmol, 1 equiv.), **2a** (3 equiv.), and base (5 equiv.) in solvent (1 mL). ^b^ NMR yield determined by employing 4-methoxyanisole as internal standard. ^c^ Isolated yield.

**Table 2:** Substrate scope of aryl sulfonium salt ^a^.

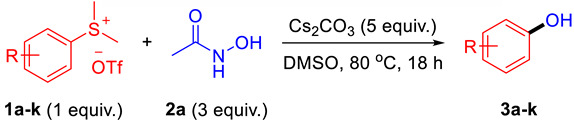			
Entry	Product		Yield (%) ^b^
1	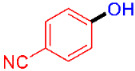	**3a**	86
2	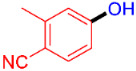	**3b**	49
3	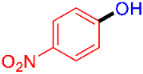	**3c**	95
4	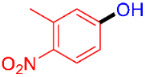	**3d**	79
5	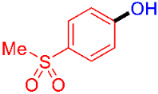	**3e**	81
6	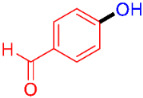	**3f**	61
7	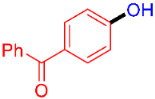	**3g**	56
8	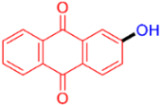	**3h**	73
9	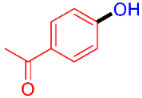	**3i**	51
10	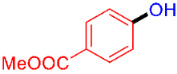	**3j**	54
11	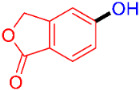	**3k**	70

^a^ The reactions were conducted at 80 °C for 18 h by employing aryl sulfonium salt **1a**–**k** (0.5 mmol), acetohydroxamic acid **2a** (1.5 mmol), and Cs_2_CO_3_ (2.5 mmol) in DMSO (2 mL). ^b^ Isolated yield.

**Table 3:** Optimization of reaction conditions ^a^.

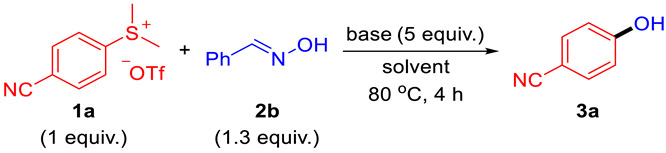			
Entry	Base	Solvent	Yield (%) ^b^
1	Cs_2_CO_3_	DMSO	58
2	Cs_2_CO_3_	NMP	63
3	Cs_2_CO_3_	MeCN	62
4	Cs_2_CO_3_	1,4-dioxane	54
5	Cs_2_CO_3_	toluene	15
6	Cs_2_CO_3_	THF	56
7	Cs_2_CO_3_	DMF	67
8	DBU	DMF	16
9	DABCO	DMF	<5
10	DIPEA	DMF	<5
11	CsF	DMF	57
12	NaOAc	DMF	<5
13	K_3_PO_4_	DMF	56
14	NaHCO_3_	DMF	<5
15	KHCO_3_	DMF	22
16	Na_2_CO_3_	DMF	<5
17	K_2_CO_3_	DMF	47
18	*^t^*BuOK	DMF	66
19	LDA	DMF	58
20	Cs_2_CO_3_	DMF	74 ^c^ (70) ^d^

^a^ Unless otherwise specified, the reactions were conducted at 80 °C for 4 h by employing **1a** (0.5 mmol, 1 equiv.), **2b** (1.3 equiv.), and base (5 equiv.) in solvent (2 mL). ^b^ NMR yield determined by employing 4-methoxyanisole as internal standard. ^c^ Using 1.5 equiv. of **2b**. ^d^ Isolated yield.

**Table 4:** Screening of various oximes ^a^.

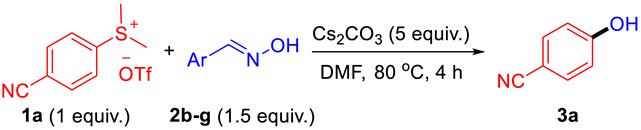			
Entry	Oxime		Yield (%) ^b^
1	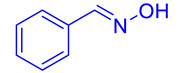	**2b**	74
2	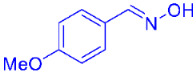	**2c**	41
3	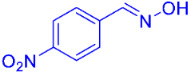	**2d**	82
4	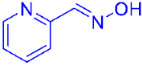	**2e**	45
5	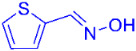	**2f**	38
6	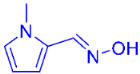	**2g**	89 (87) ^c^

^a^ The reactions were conducted at 80 °C for 4 h by employing **1a** (0.5 mmol, 1 equiv.), **2b**–**g** (1.5 equiv.), and Cs_2_CO_3_ (5 equiv.) in DMF (2 mL). ^b^ NMR yield determined by employing 4-methoxyanisole as internal standard. ^c^ Isolated yield.

**Table 5:** Substrate scope of aryl sulfonium salt ^a^.

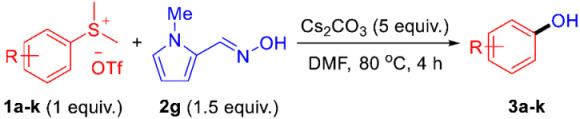			
Entry	Product		Yield (%) ^b^
1	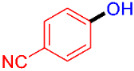	**3a**	87
2	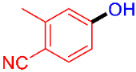	**3b**	59
3	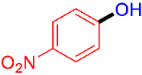	**3c**	95
4	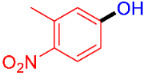	**3d**	55
5	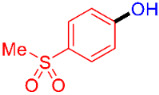	**3e**	79
6	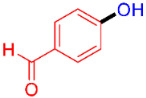	**3f**	52
7	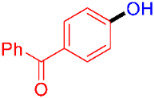	**3g**	53
8	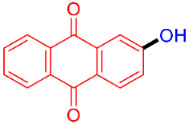	**3h**	68
9	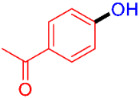	**3i**	60
10	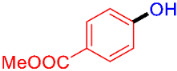	**3j**	47
11	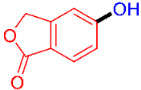	**3k**	60

^a^ The reactions were conducted at 80 °C for 4 h by employing aryl sulfonium salt **1a**–**k** (0.5 mmol), **2g** (0.75 mmol), and Cs_2_CO_3_ (2.5 mmol) in DMF (2 mL). ^b^ Isolated yield.

## Data Availability

Data are contained within the article and Supplementary Materials.

## Supplementary Materials

The following supporting information can be downloaded at: https://www.mdpi.com/article/10.3390/molecules29040831/s1.
