# Supplementary material for: Hydroxylation of Aryl Sulfonium Salts for Phenol Synthesis under Mild Reaction Conditions
Source: Molecules. 2024 Feb 13;29(4):831. doi: 10.3390/molecules29040831 (PMC10891898; doi:10.3390/molecules29040831)

Supporting Information

Table of Contents

Commercial suppliers and the purity of reagents… .... page S3

Abbreviations list… .... page S4

Figure S1. ^{1}H NMR spectrum of **3a** (400 MHz, $CDCl_3$).. page S5

Figure S2. ^{13}C NMR spectrum of **3a** (100 MHz, $CDCl_3$)… .... page S5

Figure S3. ^{1}H NMR spectrum of **3b** (400 MHz, $CDCl_3$). .... page S6

Figure S4. ^{13}C NMR spectrum of **3b** (100 MHz, $CDCl_3$).. page S6

Figure S5. ^{1}H NMR spectrum of **3c** (400 MHz, $CDCl_3$).. page S7

Figure S6. ^{13}C NMR spectrum of **3c** (100 MHz, $CDCl_3$)... page S7

Figure S7. ^{1}H NMR spectrum of **3d** (400 MHz, DMSO-d_6)… .... page S8

Figure S8. ^{13}C NMR spectrum of **3d** (100 MHz, DMSO-d_6)… .... page S8

Figure S9. ^{1}H NMR spectrum of **3e** (400 MHz, $CDCl_3$) .... page S9

Figure S10. ^{13}C NMR spectrum of **3e** (100 MHz, $CDCl_3$)… .... page S9

Figure S11. ^{1}H NMR spectrum of **3f** (400 MHz, $CDCl_3$).. page S10

Figure S12. ^{13}C NMR spectrum of **3f** (100 MHz, $CDCl_3$).. page S10

Figure S13. ^{1}H NMR spectrum of **3g** (400 MHz, $CDCl_3$)..page S11

Figure S14. ^{13}C NMR spectrum of **3g** (100 MHz, $CDCl_3$)...page S11

Figure S15. ^{1}H NMR spectrum of **3h** (400 MHz, DMSO-d_6)… .... page S12

Figure S16. ^{13}C NMR spectrum of **3h** (100 MHz, DMSO-d_6)… .... page S12

Figure S17. ^{1}H NMR spectrum of **3i** (400 MHz, $CDCl_3$).. page S13

Figure S18. ^{13}C NMR spectrum of **3i** (100 MHz, $CDCl_3$)… .... page S13

Figure S19. ^{1}H NMR spectrum of **3j** (400 MHz, $CDCl_3$)… .... page S14

Figure S20. ^{13}C NMR spectrum of **3j** (100 MHz, $CDCl_3$)... page S14

Figure S21. ^{1}H NMR spectrum of **3k** (400 MHz, DMSO-d_6)… .... page S15

Figure S22. ^{13}C NMR spectrum of **3k** (100 MHz, DMSO-d_6)… .... page S15

Figure S23. HRMS spectrum of **3a**.. page S16

Figure S24. HRMS spectrum of **3b** .... page S16

Figure S25. HRMS spectrum of **3c**... page S17

Figure S26. HRMS spectrum of **3d** .... page S17

Figure S27. HRMS spectrum of **3e**... page S18

Figure S28. HRMS spectrum of **3f** .... page S18

Figure S29. HRMS spectrum of **3g** .... page S19

Figure S30. HRMS spectrum of **3h**.. page S19

Figure S31. HRMS spectrum of **3i** .... page S20

Figure S32. HRMS spectrum of **3j**... page S20

Figure S33. HRMS spectrum of **3k**.. page S21

Commercial suppliers and the purity of reagents

Name	CAS No.	Purity	Supplier
4-(methylthio)benzonitrile	21382-98-9	99%	Adamas
methyl(4-nitrophenyl)sulfane	701-57-5	99%	Adamas
methyl(3-methyl-4-nitrophenyl)sulfane	90972-20-6	98%	Adamas
2-methyl-4-(methylthio)benzonitrile	1190948-25-4	99%	Matrix
methyl 4-(methylthio)benzoate	3795-79-7	99%	Sigma-Aldrich
1-(4-(methylthio)phenyl)ethan-1-one	1778-09-2	99%	Adamas
4-(methylthio)benzaldehyde	3446-89-7	98%	Adamas
methyl(4-(methylsulfonyl)phenyl)sulfane	52323-93-0	98%	Adamas
(4-(methylthio)phenyl)(phenyl)methanone	23405-48-3	98%	HWRK Chem
2-(methylthio)anthracene-9,10-dione	78507-47-8	98%	Adamas
methyl trifluoromethanesulfonate	333-27-7	98%	Adamas
4-methoxybenzaldehyde	123-11-5	99%	Adamas
4-nitrobenzaldehyde	555-16-8	99%	Adamas
picolinaldehyde	1121-60-4	98%	Adamas
thiophene-2-carbaldehyde	98-03-3	99%	Adamas
1-methyl-1*H*-pyrrole-2-carbaldehyde	1192-58-1	98%	Macklin
acetohydroxamic acid	546-88-3	98%	Macklin
benzaldoxime	932-90-1	99%	Adamas
K_2CO_3	534-17-8	99%	Bidepharm
DBU	6674-22-2	99%	Heowns
DABCO	280-57-9	98%	Bidepharm
CsF	13400-13-0	99%	Meryer
NaOAc	127-09-3	99%	Heowns
K_3PO_4	7778-53-2	97%	Heowns
Na_2CO_3	5968-11-6	98%	Adamas
K_2CO_3	584-08-7	99%	Macklin
KOH	1310-58-3	95%	Meryer
^{t}BuOK	865-47-4	99%	Heowns
LDA	4111-54-0	2 M in THF	J&K Scientific
DIPEA	7087-68-5	99%	Adamas
$NaHCO_3$	144-55-8	99%	Heowns
$KHCO_3$	298-14-6	98%	Leyan
$NH_2OH{\cdot}HCl$	5470-11-1	99%	Adamas
MeOH	67-56-1	99.8%	J&K Scientific
DCE	107-06-2	99.5%	J&K Scientific
DMSO	67-68-5	99.5%	J&K Scientific
DMF	68-12-2	99.8%	J&K Scientific
1,4-dioxane	123-91-1	99.5%	J&K Scientific
toluene	108-88-3	99.8%	J&K Scientific
NMP	872-50-4	99.8%	J&K Scientific
MeCN	75-05-8	99.8%	J&K Scientific
THF	109-99-9	99.9%	J&K Scientific

Abbreviations list

Abbreviation	Full name
etc.	et cetera
TM	transition metal
DBU	1,8-diazabicyclo[5,4,0]undec-7-ene
DABCO	1,4-diaza[2,2,2]bicyclooctane
DIPEA	*N*,*N*-diisopropylethylamine
LDA	lithium diisopropylamide
DMSO	dimethyl sulfoxide
DMF	*N*,*N*-dimethylformamide
NMP	*N*-methyl-2-pyrrolidone
THF	tetrahydrofuran
DCE	1,2-dichloroethane
Equiv.	equivalent
NMR	nuclear magnetic resonance
UV	ultraviolet
FT-IR	fourier transform infrared
ESI	electro spray ionization
TLC	thin layer chromatography
TMS	tetramethylsilane
HRMS	high resolution mass spectra
brs	broad singlet
s	singlet
m	multiplet
d	doublet
t	triplet
q	quartet
dd	doublet of doublets
dt	doublet of triplets
dq	doublet of quartets
td	triplet of doublets
tt	triplet of triplets
qd	quartet of doublets
ddd	doublet of doublet of doublet
ppm	parts per million

Figure S1. ^{1}H NMR spectrum of **3a** (400 MHz, $CDCl_3$)

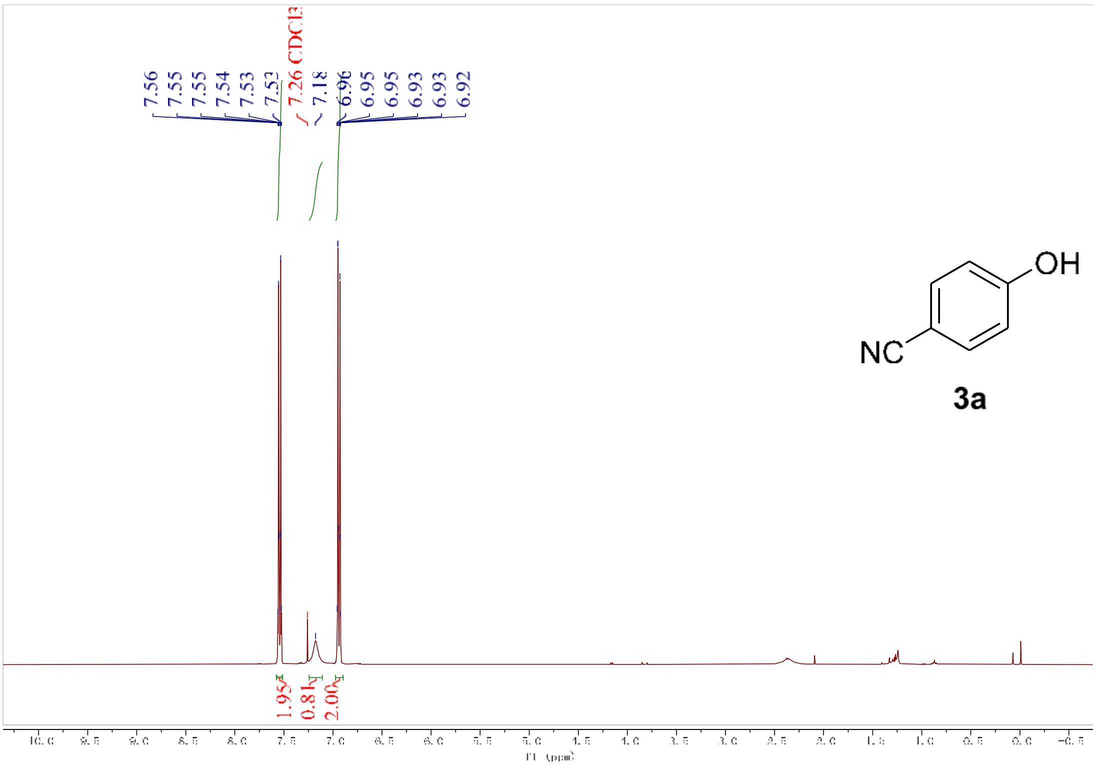

Figure S2. ^{13}C NMR spectrum of **3a** (100 MHz, $CDCl_3$)

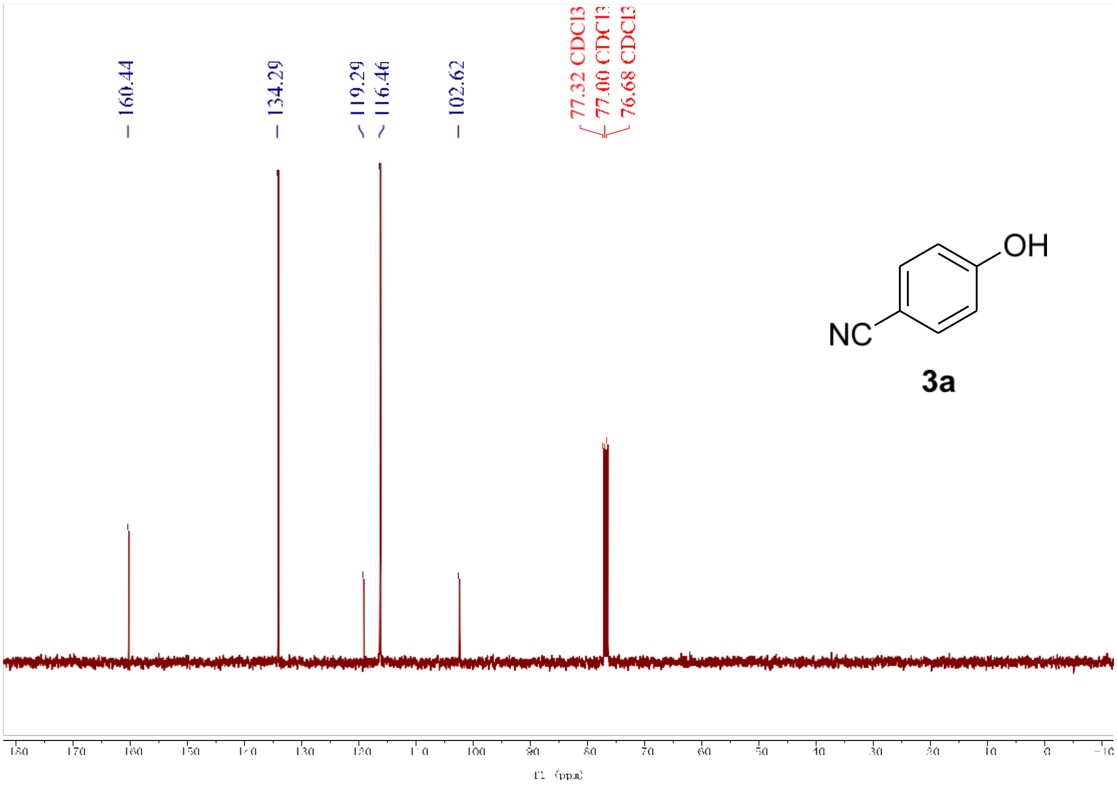

Figure S3. ^{1}H NMR spectrum of **3b** (400 MHz, $CDCl_3$)

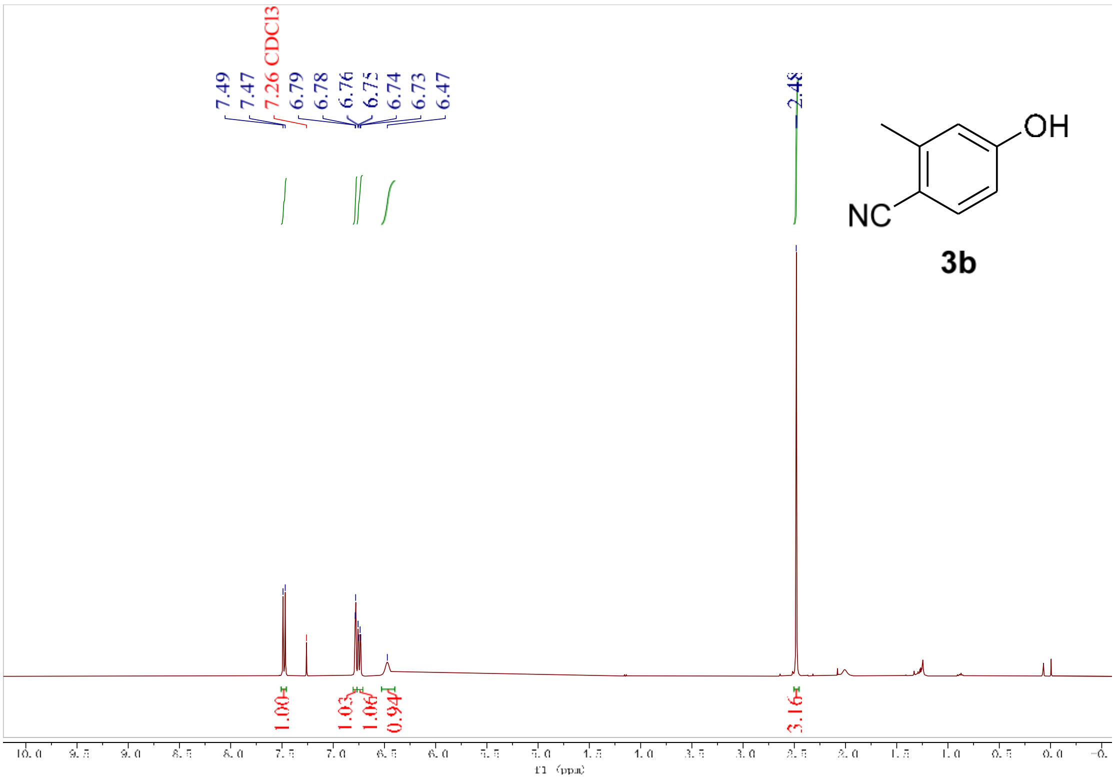

Figure S4. ^{13}C NMR spectrum of **3b** (100 MHz, $CDCl_3$)

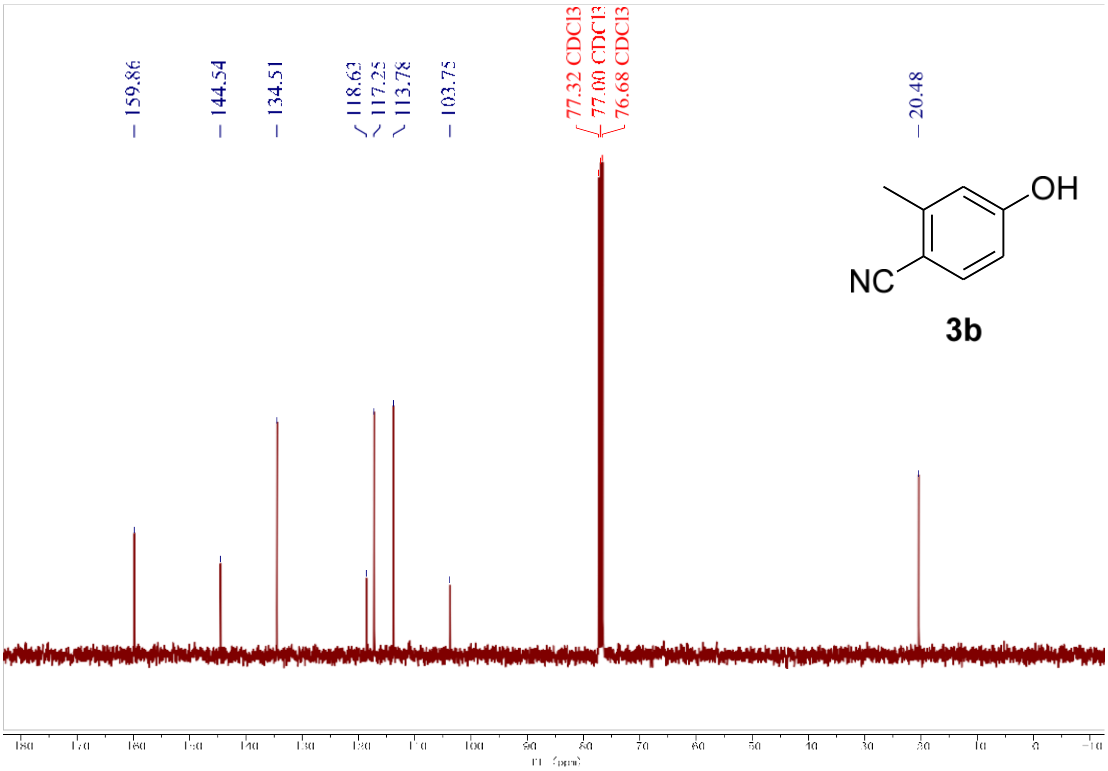

Figure S5. ^{1}H NMR spectrum of **3c** (400 MHz, $CDCl_3$)

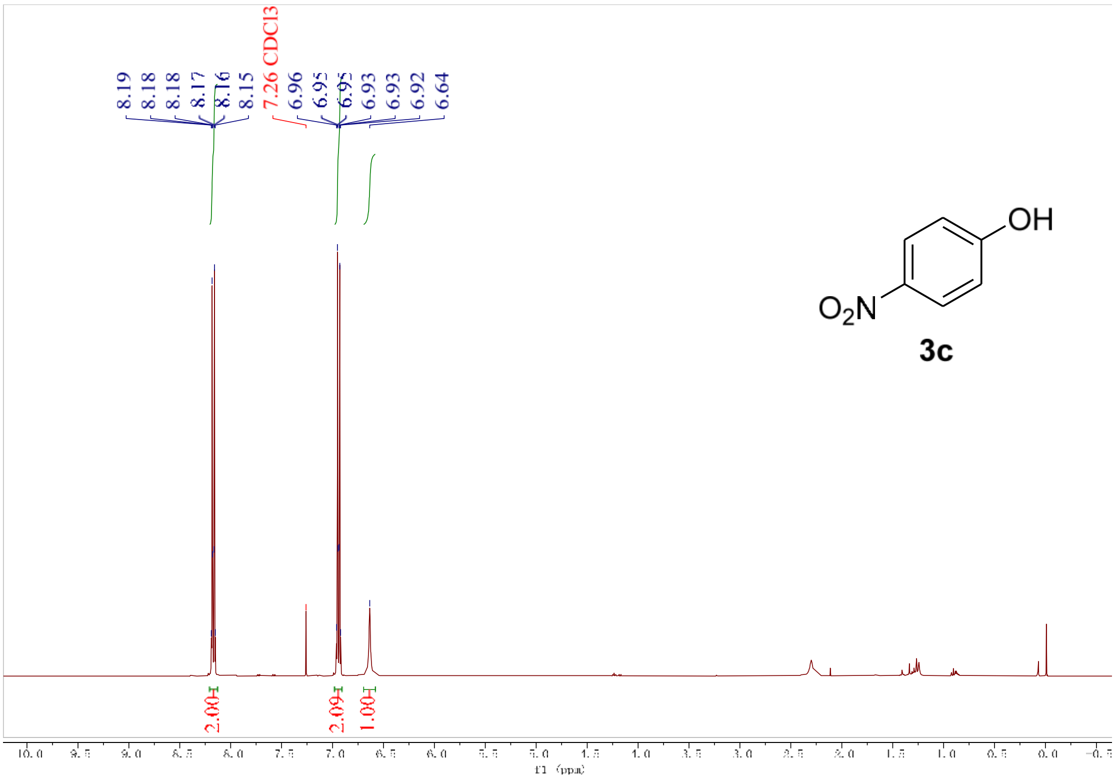

Figure S6. ^{13}C NMR spectrum of **3c** (100 MHz, $CDCl_3$)

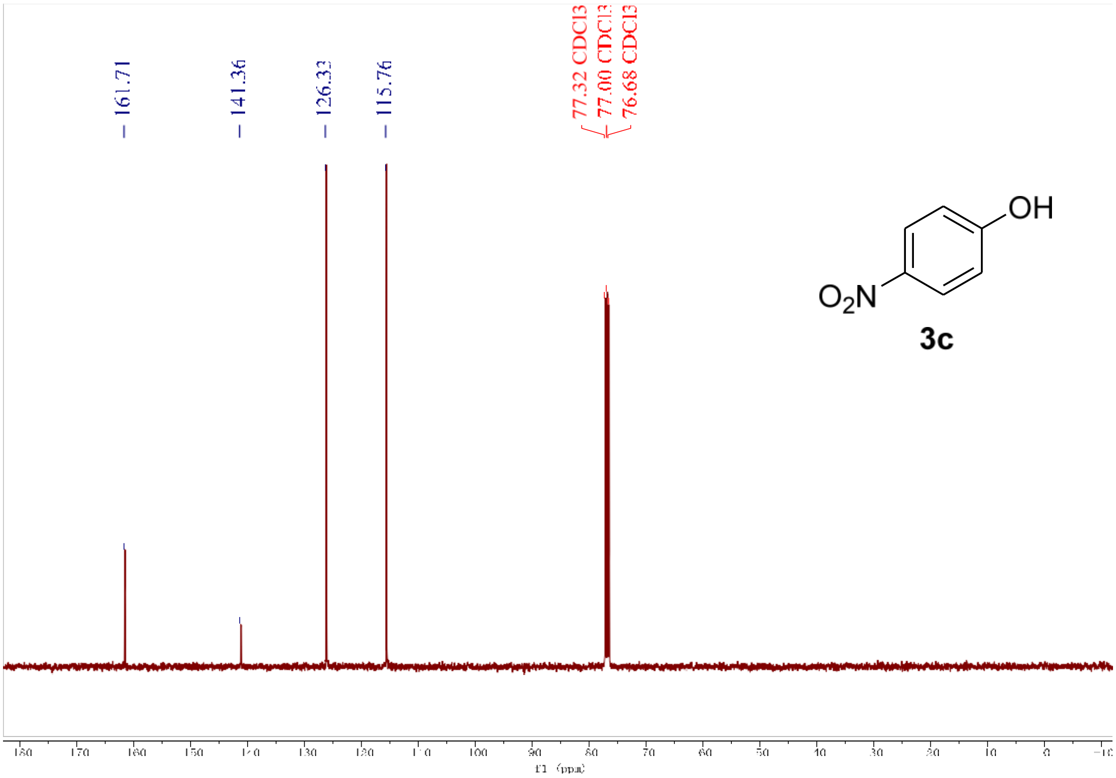

Figure S7. ^{1}H NMR spectrum of **3d** (400 MHz, DMSO-d_6)

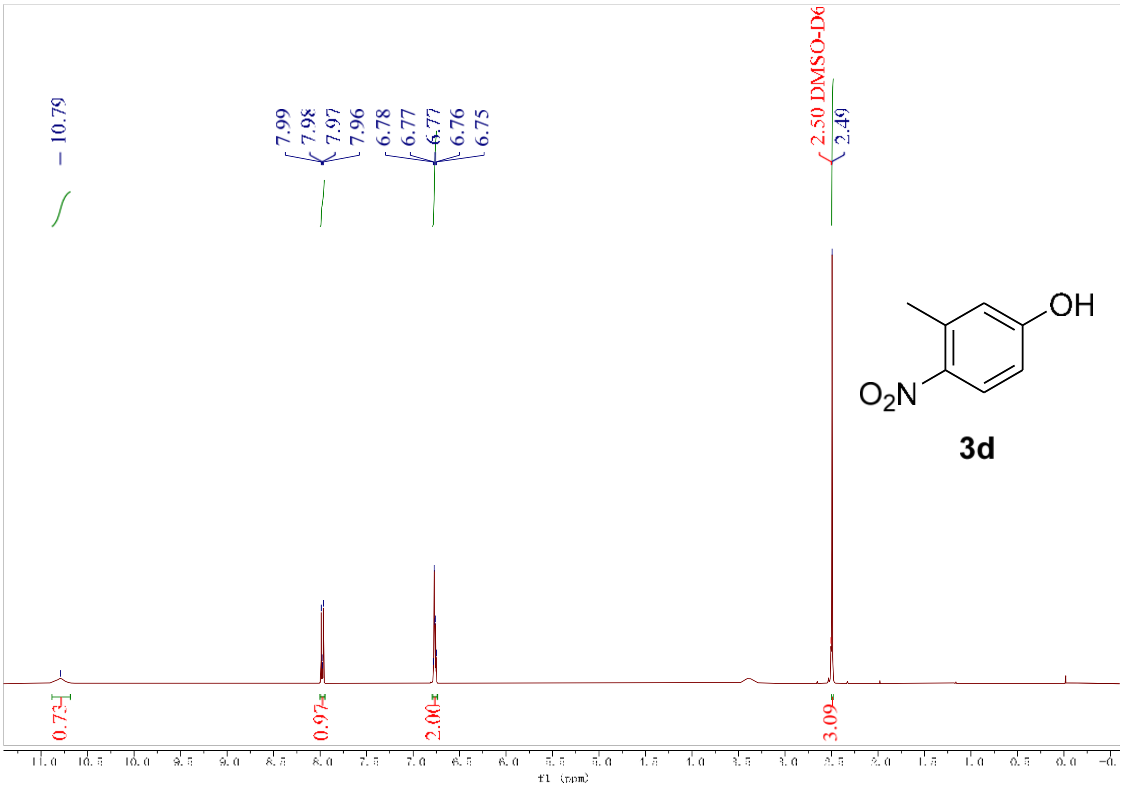

Figure S8. ^{13}C NMR spectrum of **3d** (100 MHz, DMSO-d_6)

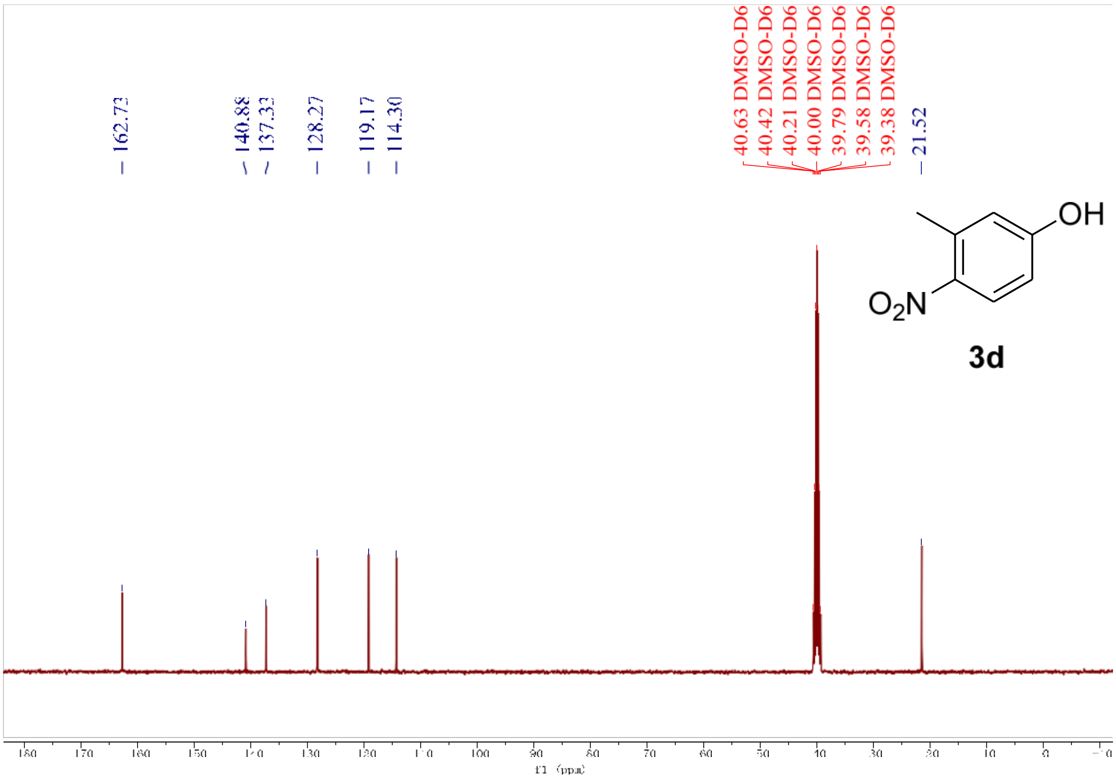

Figure S9. ^{1}H NMR spectrum of **3e** (400 MHz, $CDCl_3$)

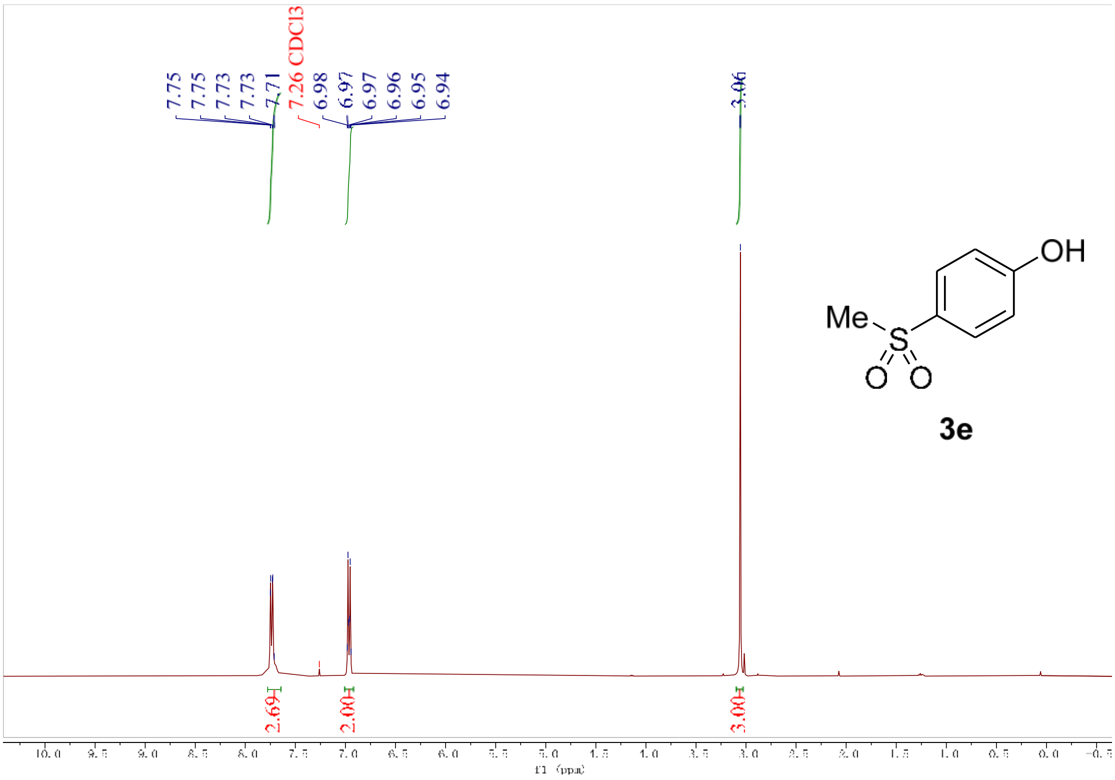

Figure S10. ^{13}C NMR spectrum of **3e** (100 MHz, $CDCl_3$)

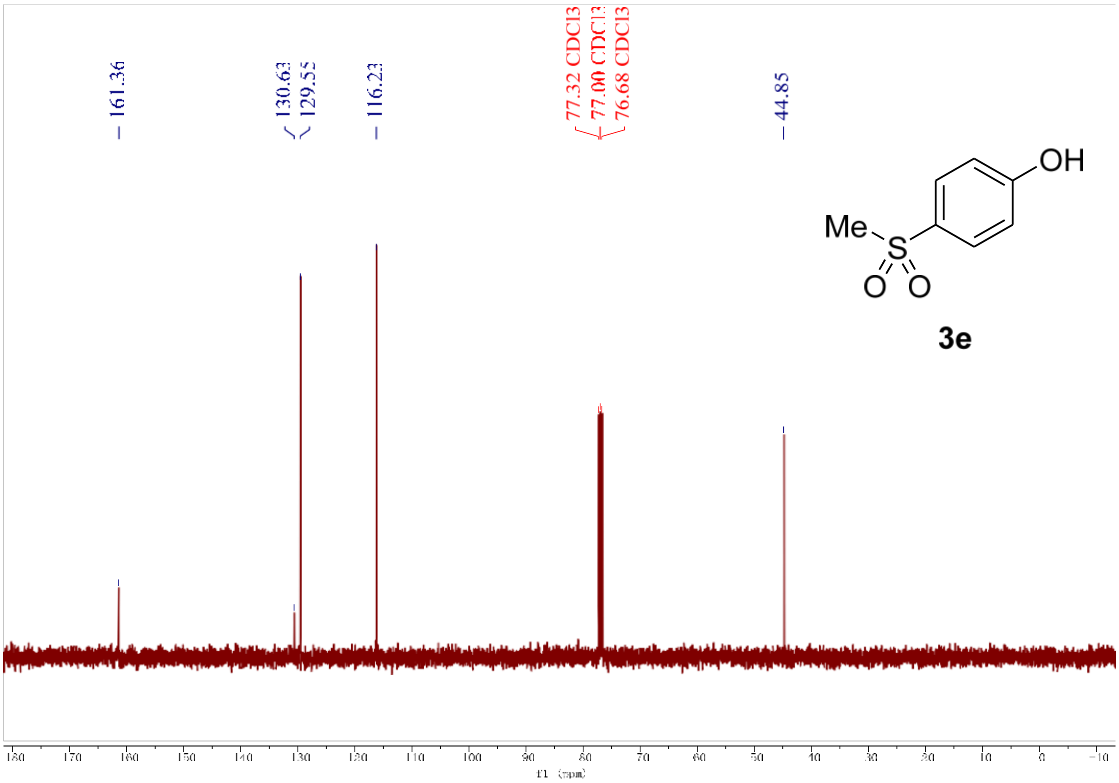

Figure S11. ^{1}H NMR spectrum of **3f** (400 MHz, $CDCl_3$)

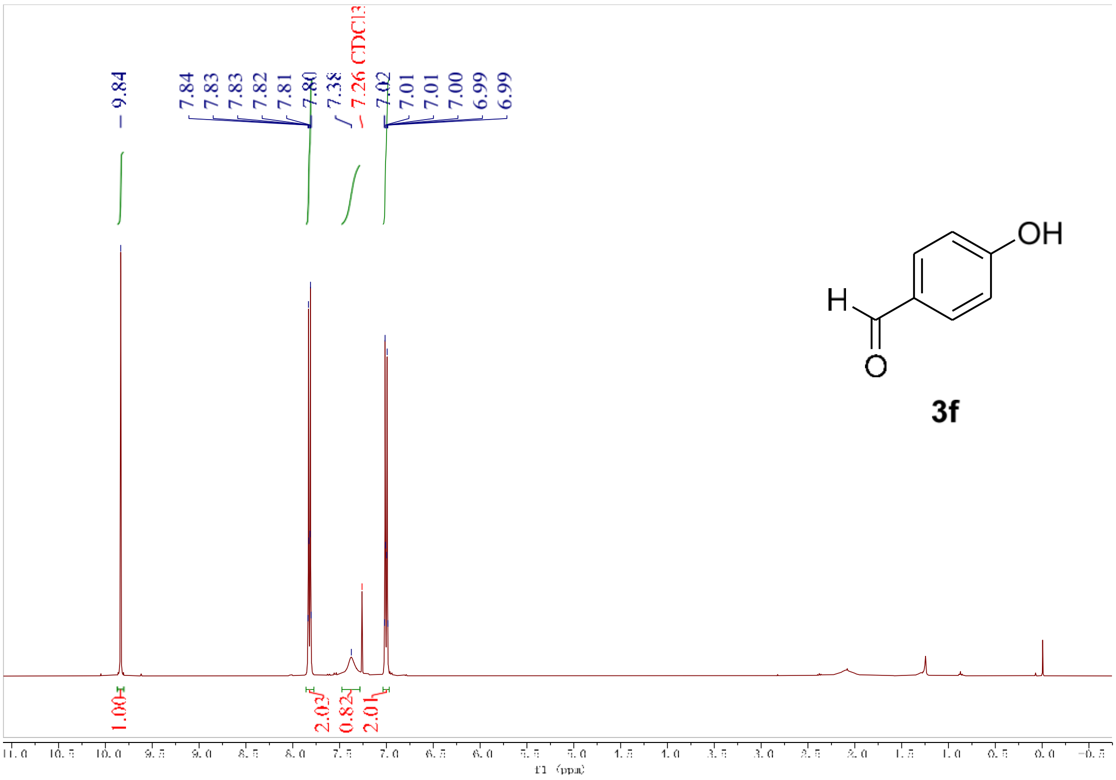

Figure S12. ^{13}C NMR spectrum of **3f** (100 MHz, $CDCl_3$)

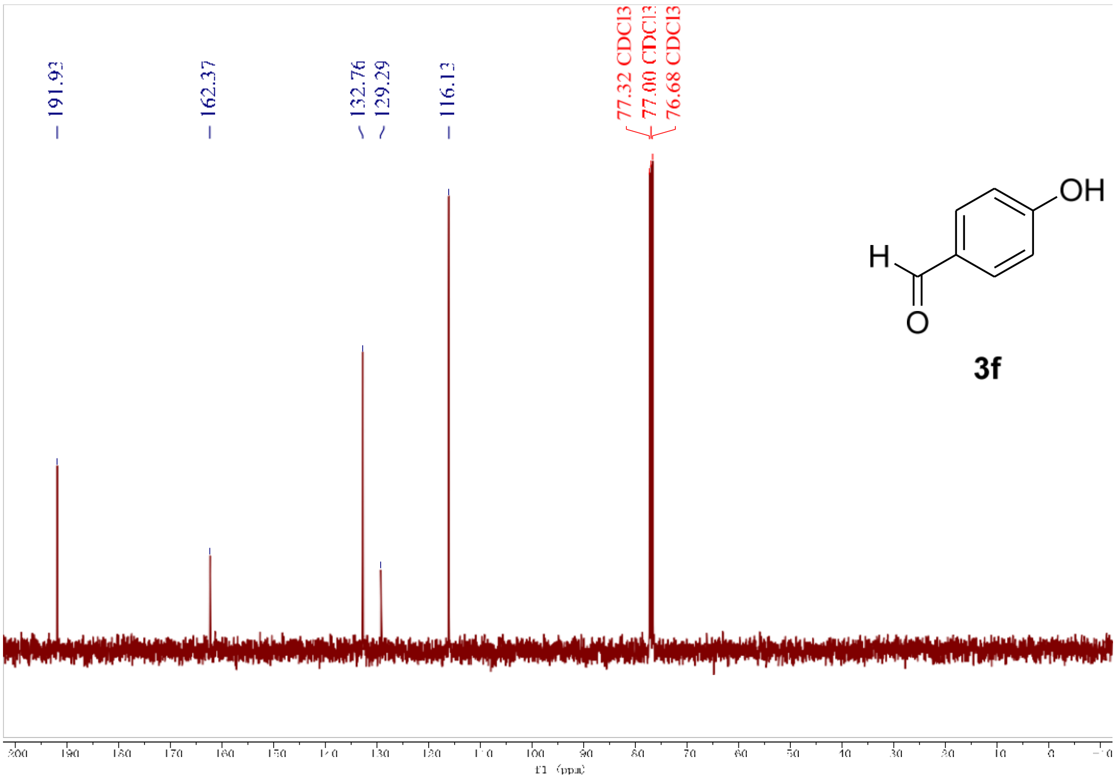

Figure S13. ^{1}H NMR spectrum of **3g** (400 MHz, $CDCl_3$)

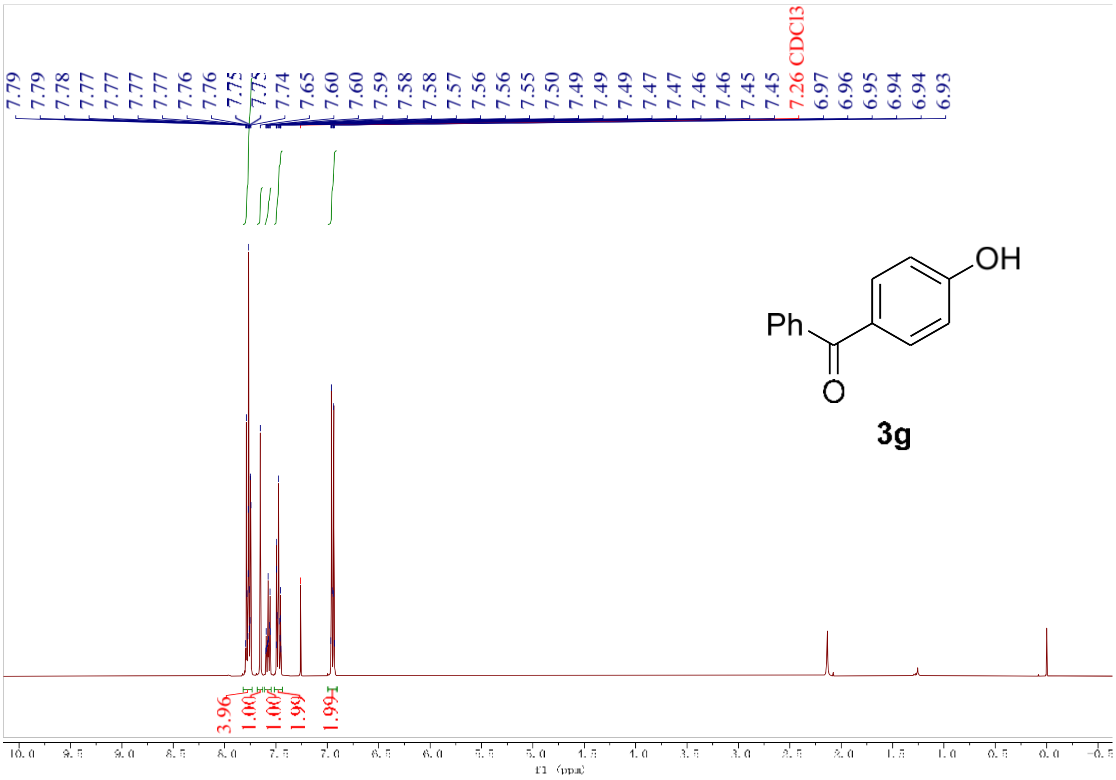

Figure S14. ^{13}C NMR spectrum of **3g** (100 MHz, $CDCl_3$)

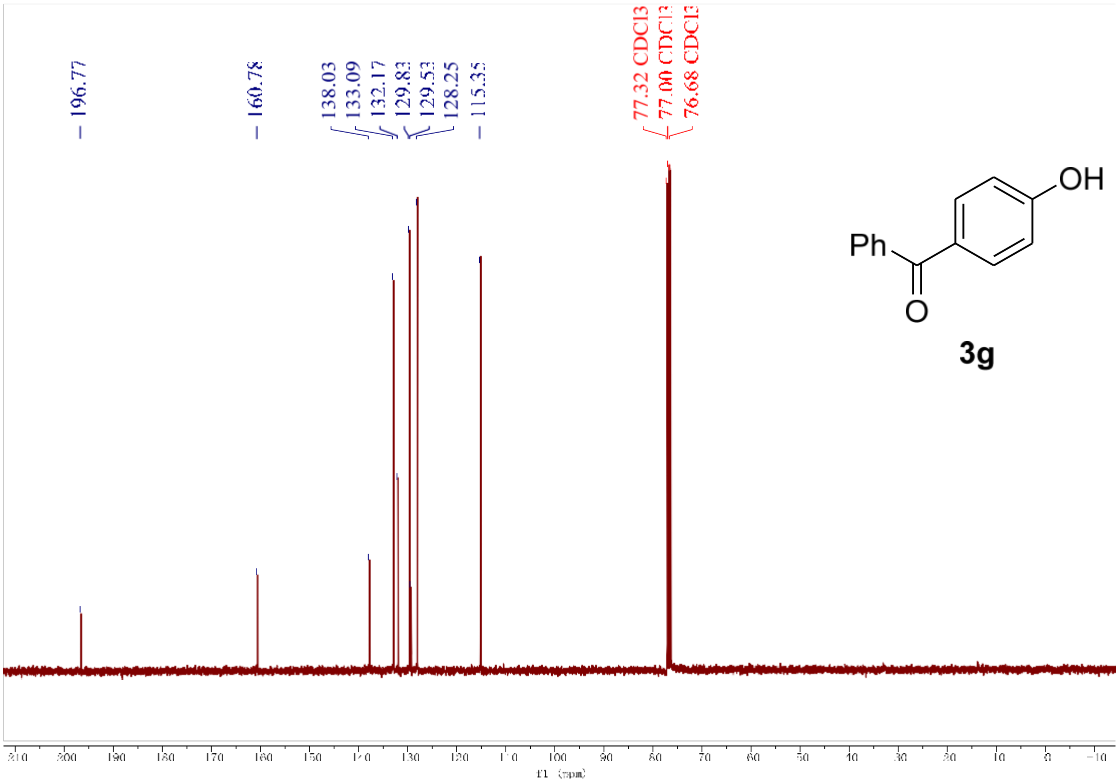

Figure S15. ^{1}H NMR spectrum of **3h** (400 MHz, DMSO-d_6)

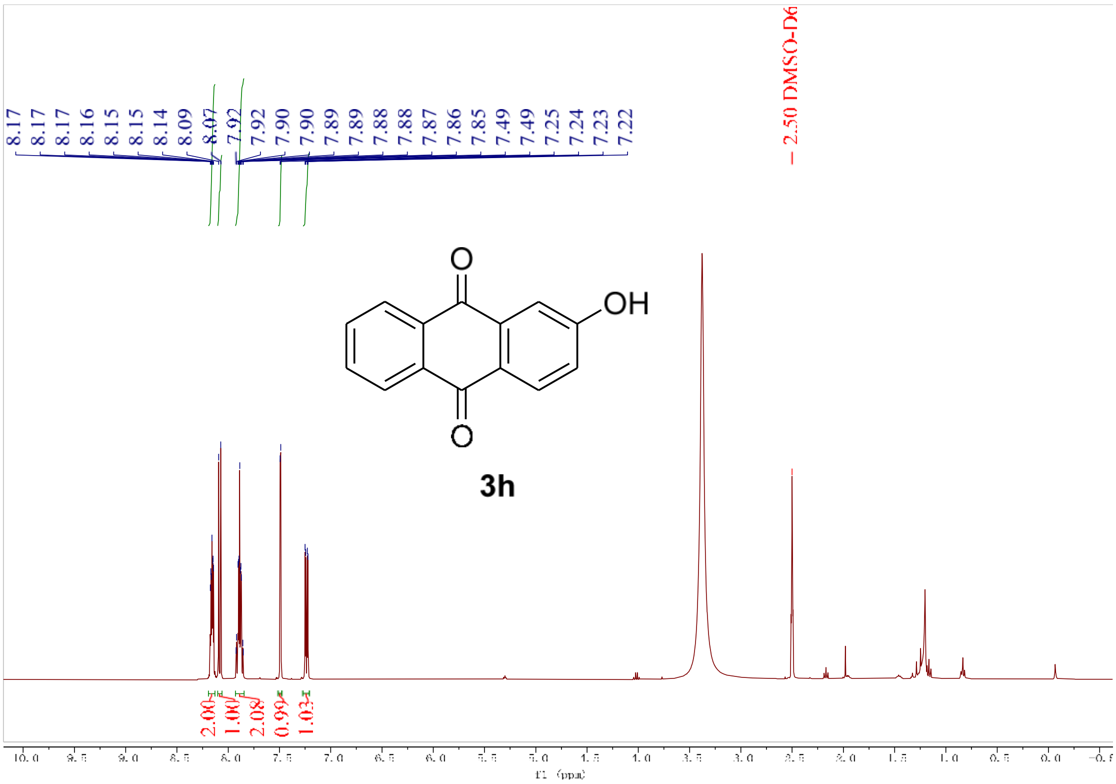

Figure S16. ^{13}C NMR spectrum of **3h** (100 MHz, DMSO-d_6)

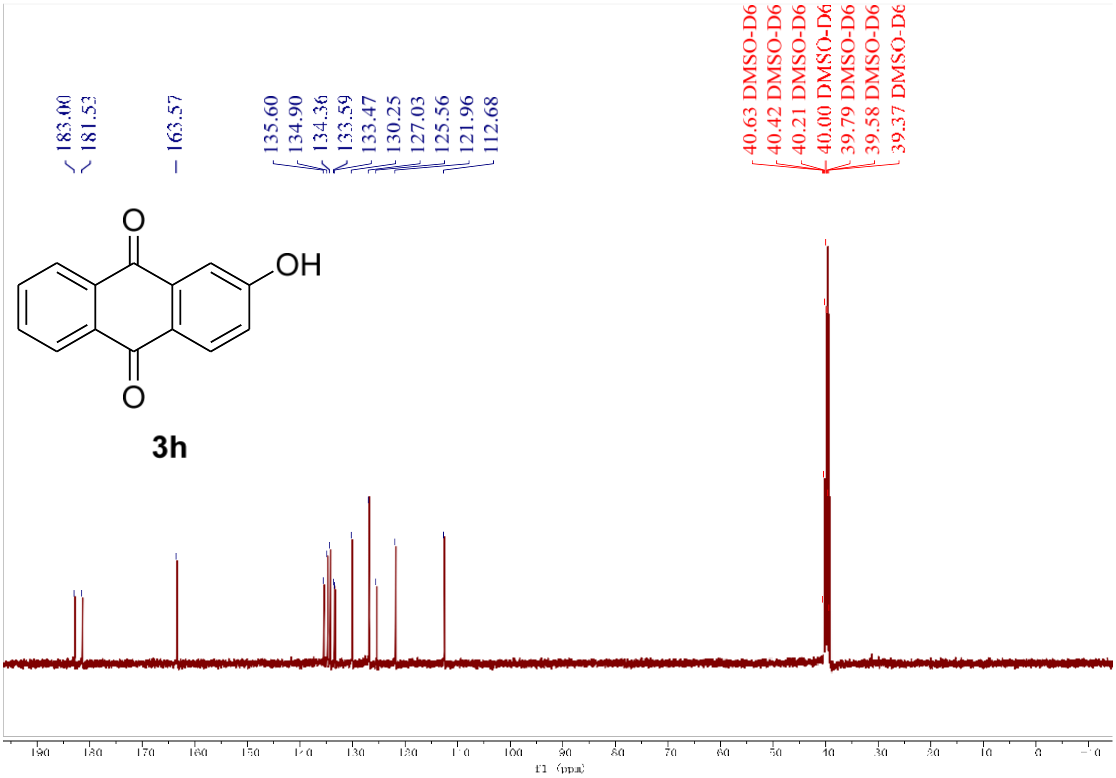

Figure S17. ^{1}H NMR spectrum of **3i** (400 MHz, $CDCl_3$)

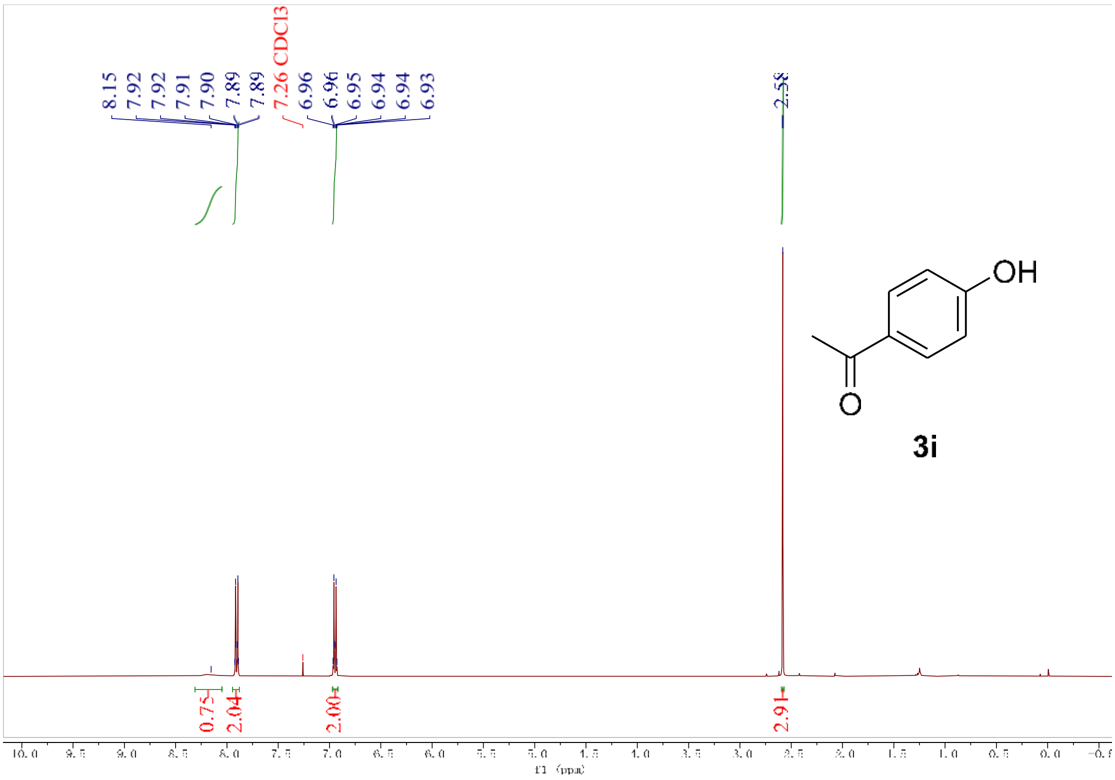

Figure S18. ^{13}C NMR spectrum of **3i** (100 MHz, $CDCl_3$)

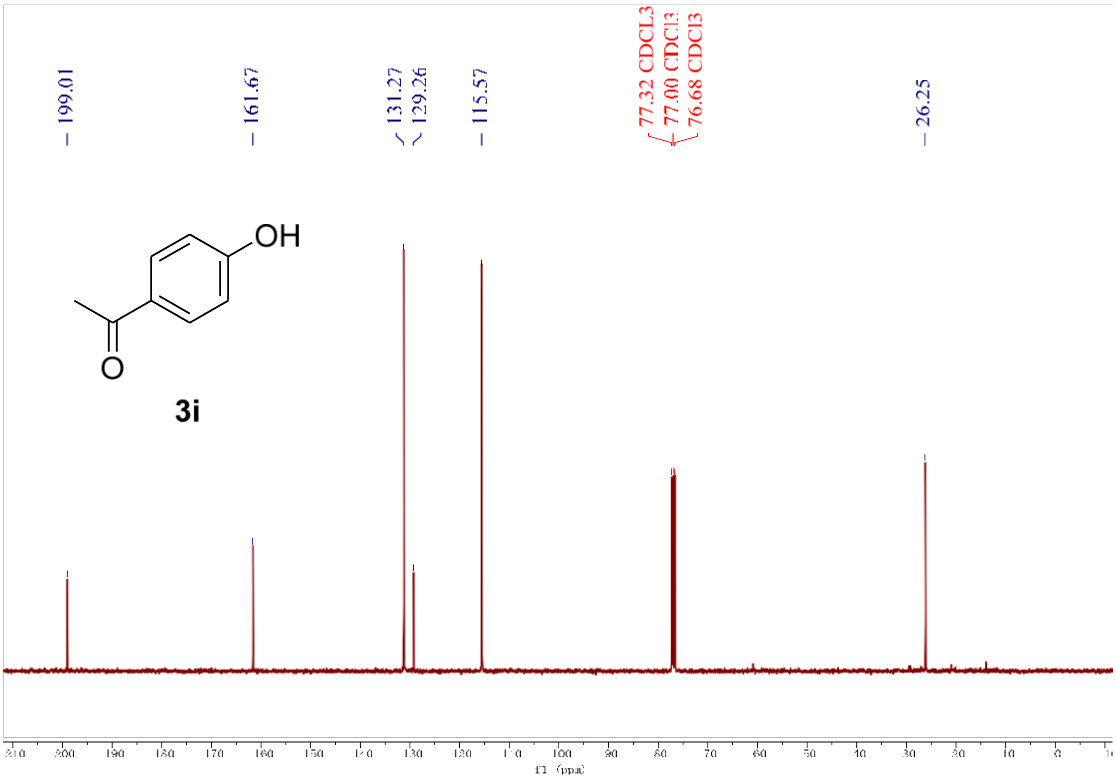

Figure S19. ^{1}H NMR spectrum of **3j** (400 MHz, $CDCl_3$)

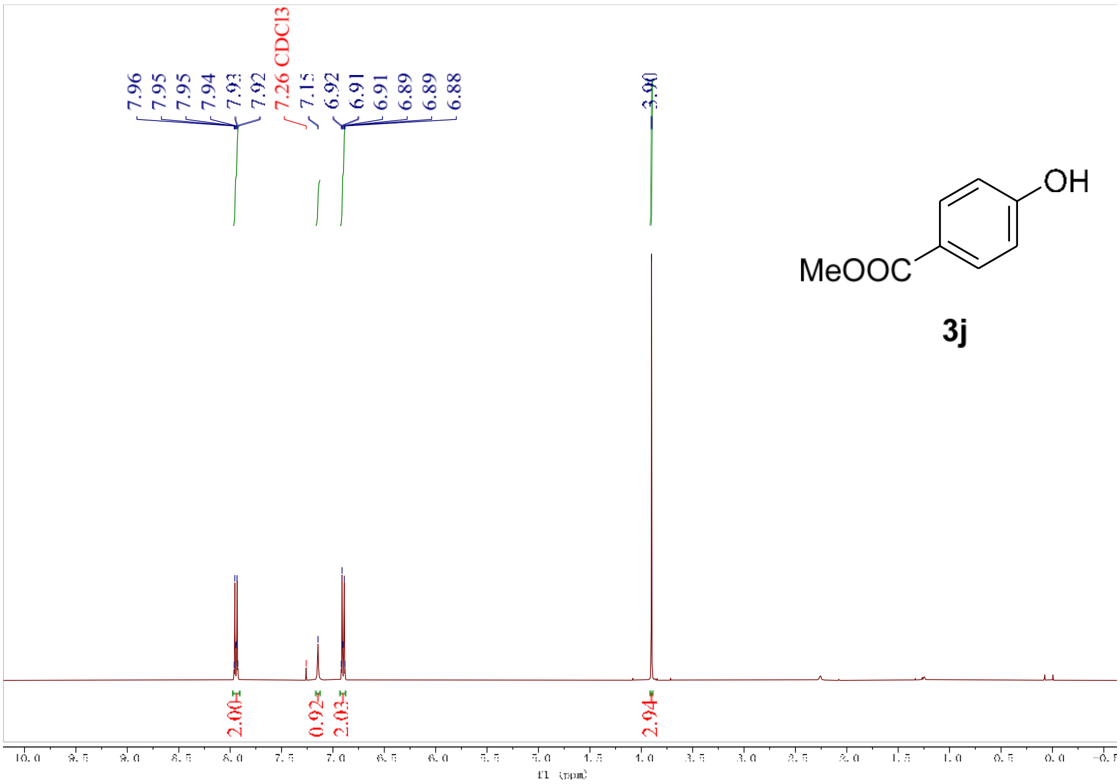

Figure S20. ^{13}C NMR spectrum of **3j** (100 MHz, $CDCl_3$)

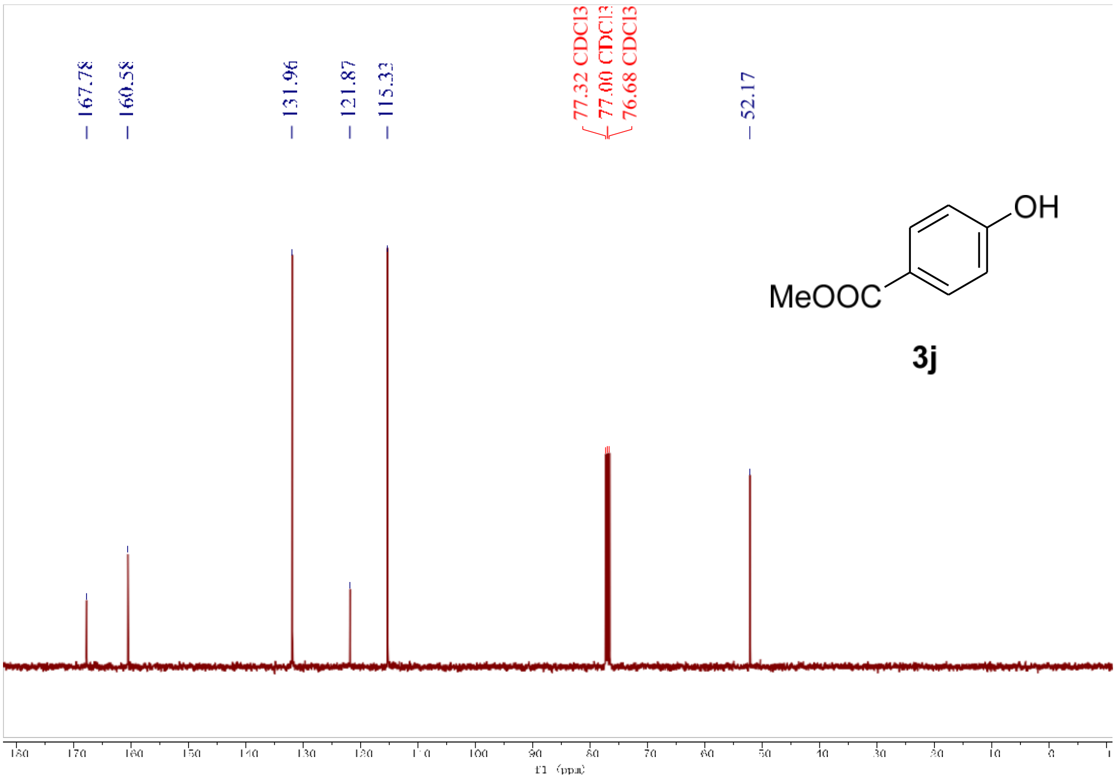

Figure S21. ^{1}H NMR spectrum of **3k** (400 MHz, DMSO-d_6)

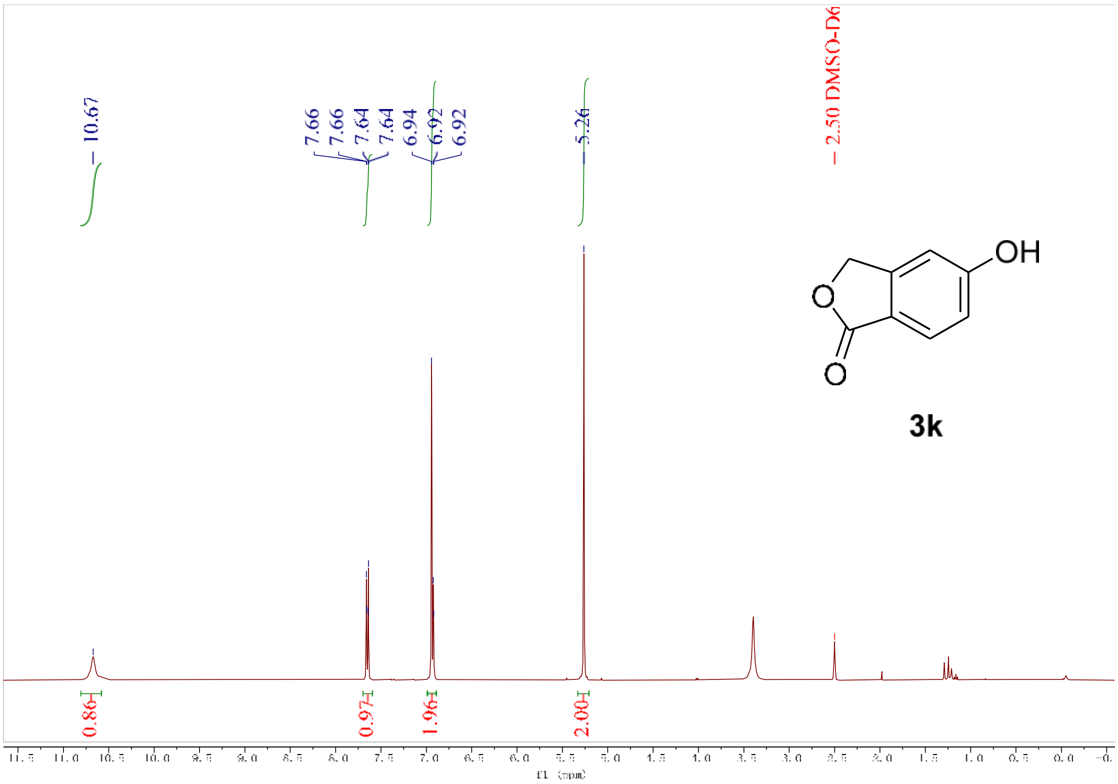

Figure S22. ^{13}C NMR spectrum of **3k** (100 MHz, DMSO-d_6)

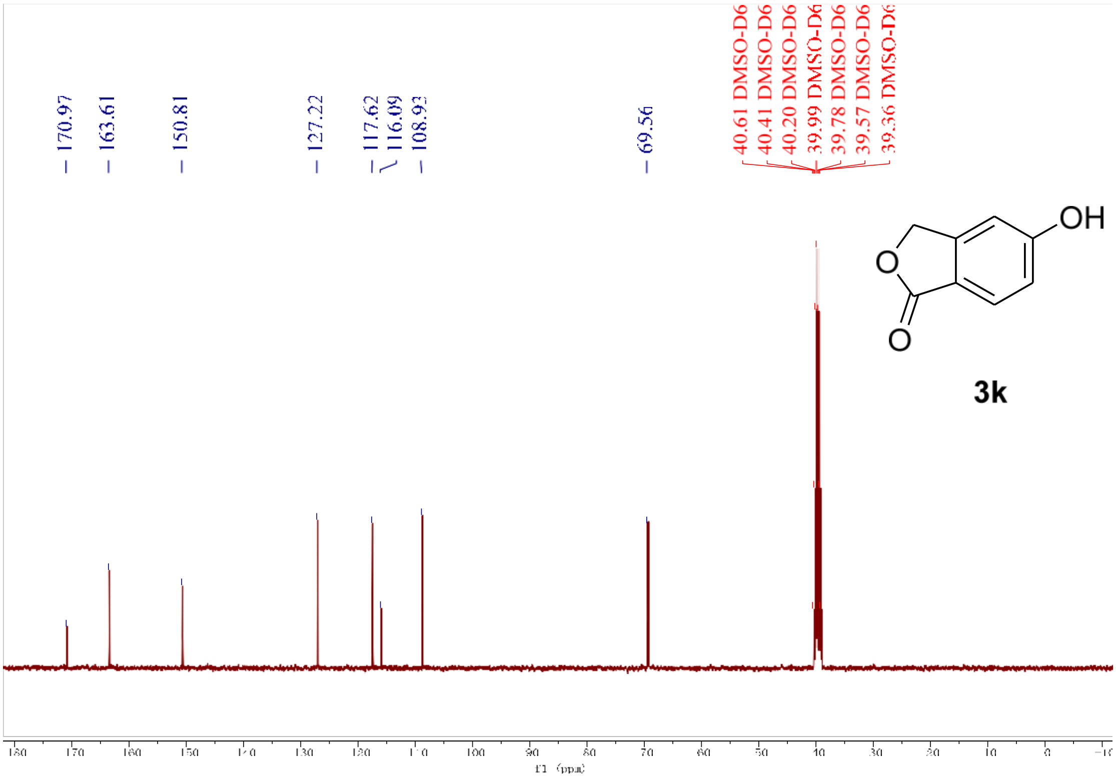

Figure S23. HRMS spectrum of **3a**

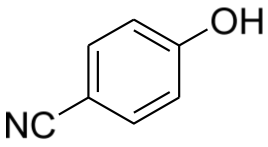

3a

HRMS (ESI, m/z): calcd for C_7H_6NO $[M+H]^+$ 120.0444, found: 120.0442.

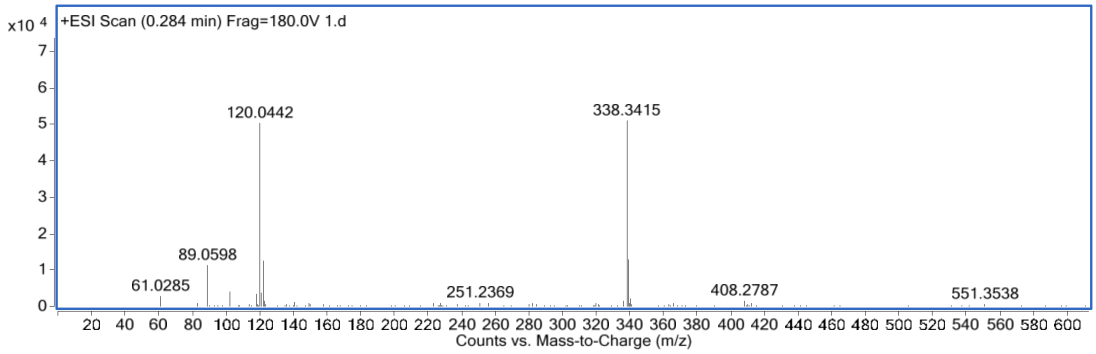

Figure S24. HRMS spectrum of **3b**

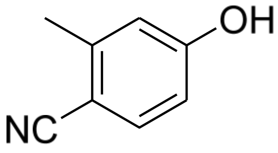

3b

HRMS (ESI, m/z): calcd for C_8H_7KNO $[M+K]^+$ 172.0159, found: 172.0170.

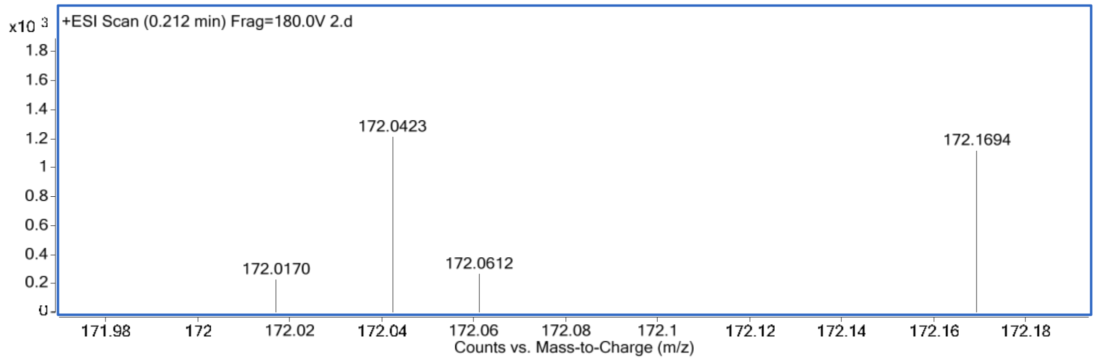

Figure S25. HRMS spectrum of **3c**

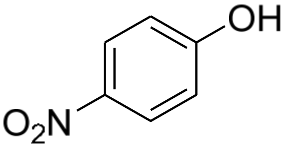

3c

HRMS (ESI, m/z): calcd for $C_6H_6NO_3$ [M+H]$^+$ 140.0342, found: 140.0353.

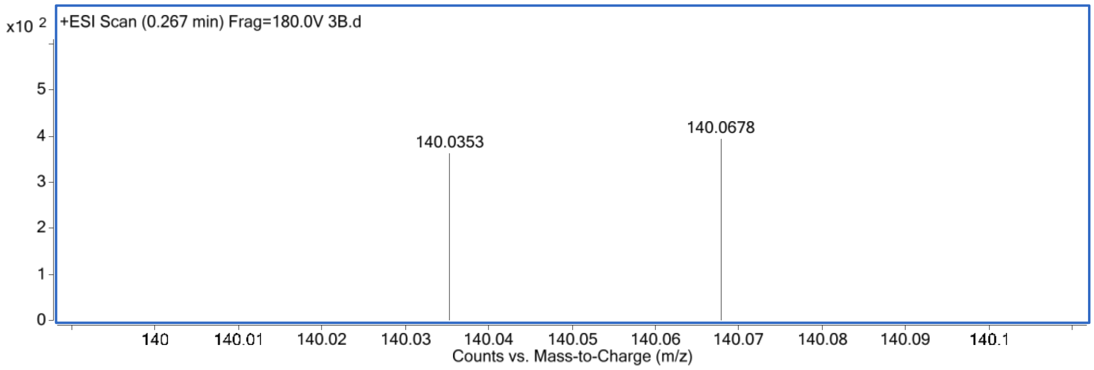

Figure S26. HRMS spectrum of **3d**

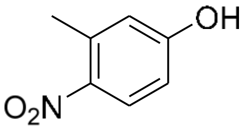

3d

HRMS (ESI, m/z): calcd for $C_7H_8NO_3$ [M+H]$^+$ 154.0499, found: 154.0498.

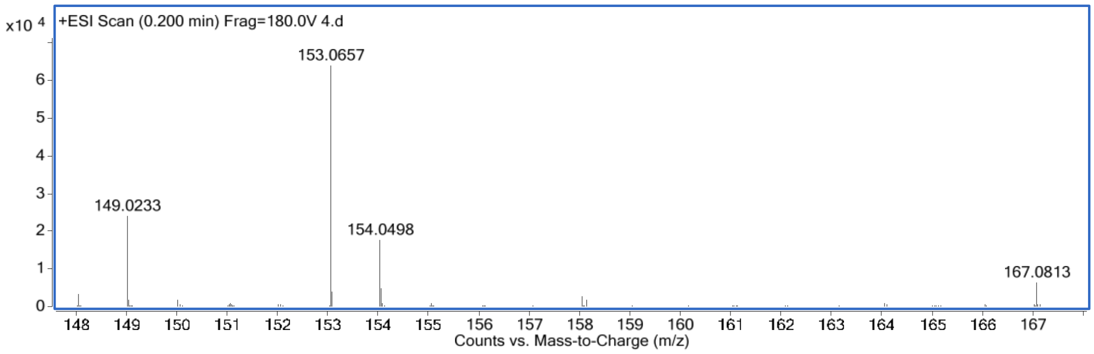

Figure S27. HRMS spectrum of **3e**

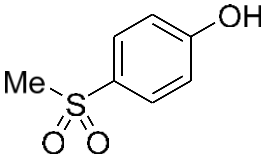

3e

HRMS (ESI, m/z): calcd for $C_7H_9O_3S$ $[M+H]^+$ 173.0267, found: 173.0266.

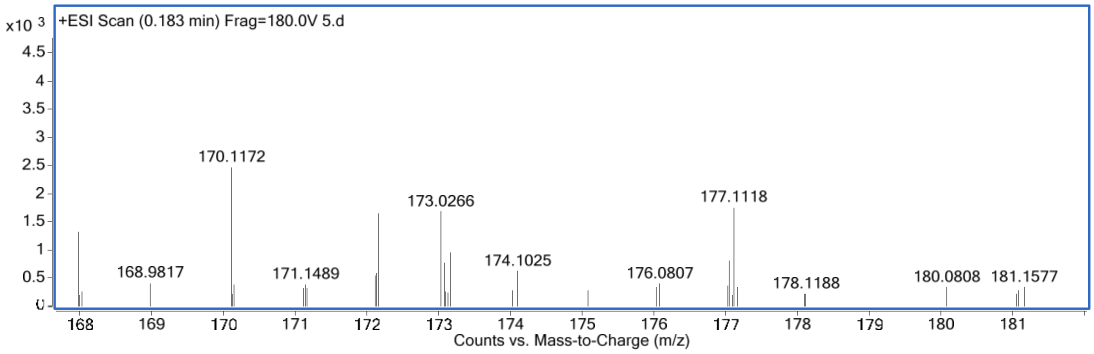

Figure S28. HRMS spectrum of **3f**

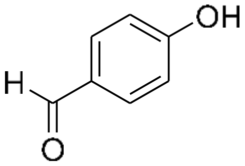

3f

HRMS (ESI, m/z): calcd for $C_7H_7O_2$ $[M+H]^+$ 123.0441, found: 123.0453.

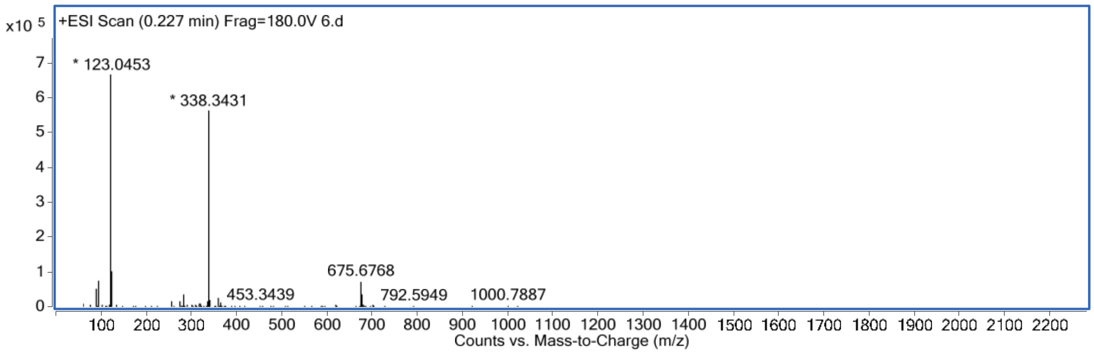

Figure S29. HRMS spectrum of **3g**

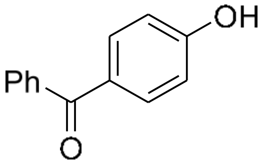

3g

HRMS (ESI, m/z): calcd for $C_{13}H_{11}O_2$ $[M+H]^+$ 199.0754, found: 199.0765.

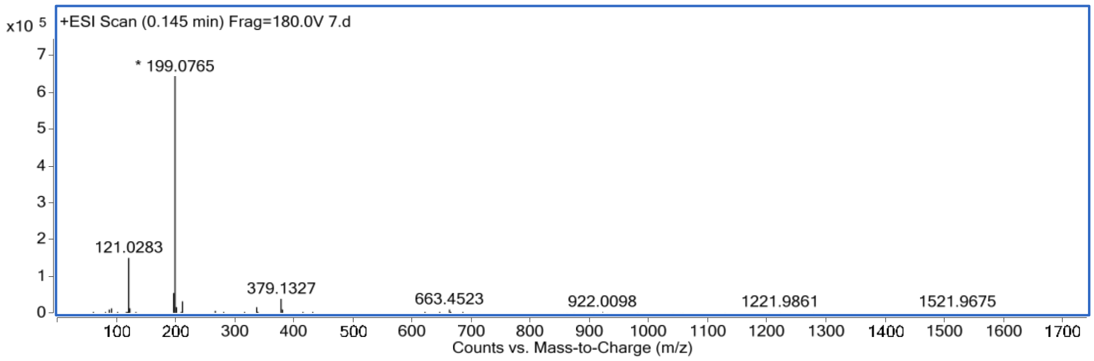

Figure S30. HRMS spectrum of **3h**

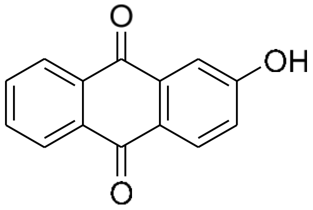

3h

HRMS (ESI, m/z): calcd for $C_{14}H_9O_3$ $[M+H]^+$ 225.0546, found: 225.0551.

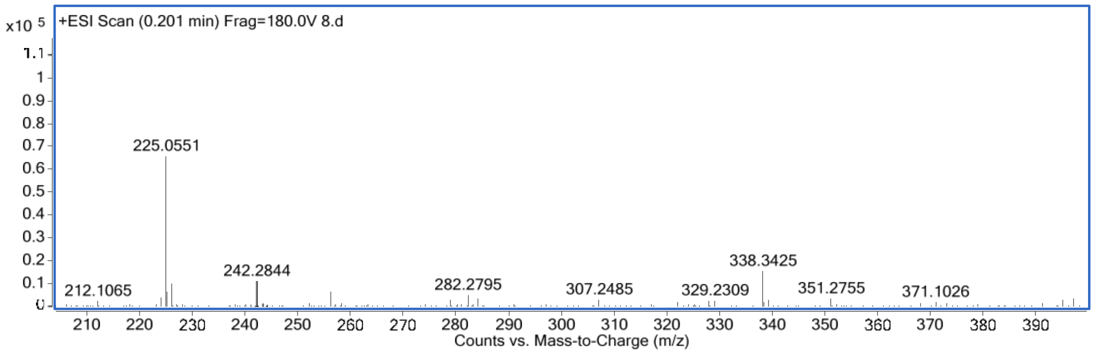

Figure S31. HRMS spectrum of **3i**

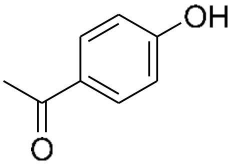

3i

HRMS (ESI, m/z): calcd for $C_8H_9O_2$ $[M+H]^+$ 137.0597, found: 137.0594.

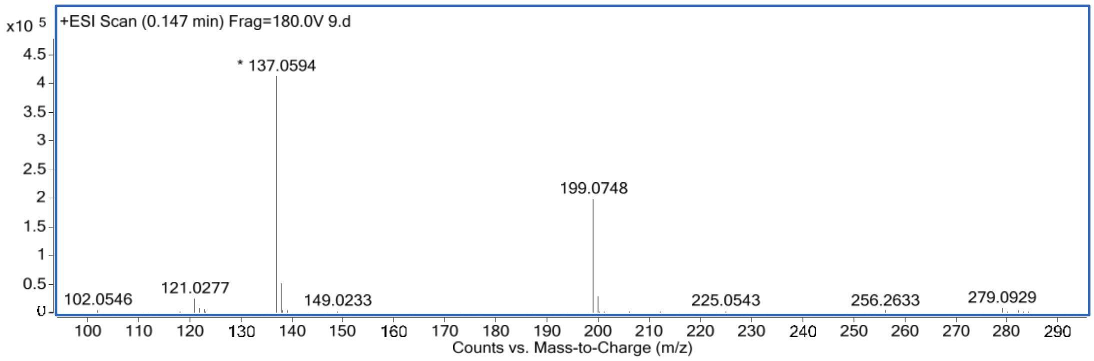

Figure S32. HRMS spectrum of **3j**

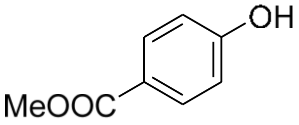

3j

HRMS (ESI, m/z): calcd for $C_8H_9O_3$ $[M+H]^+$ 153.0546, found: 153.0549.

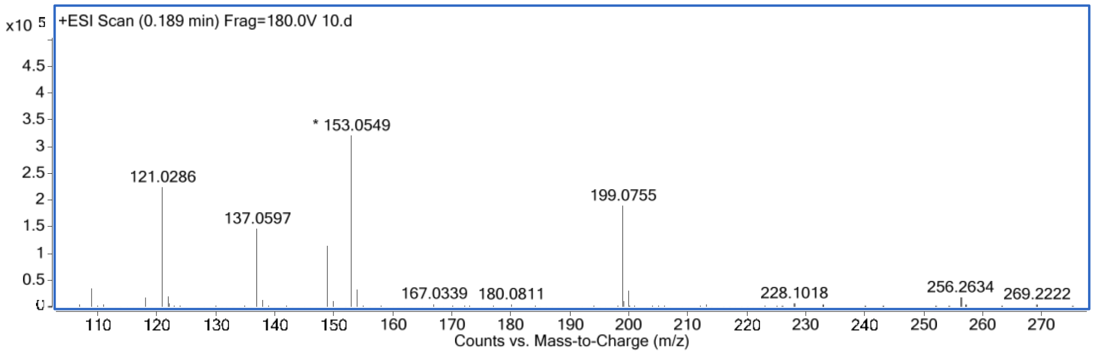

Figure S33. HRMS spectrum of **3k**

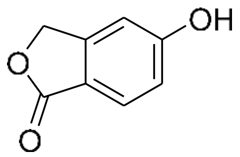

3k

HRMS (ESI, m/z): calcd for $C_8H_7O_3$ $[M+H]^+$ 151.0390, found: 151.0399.

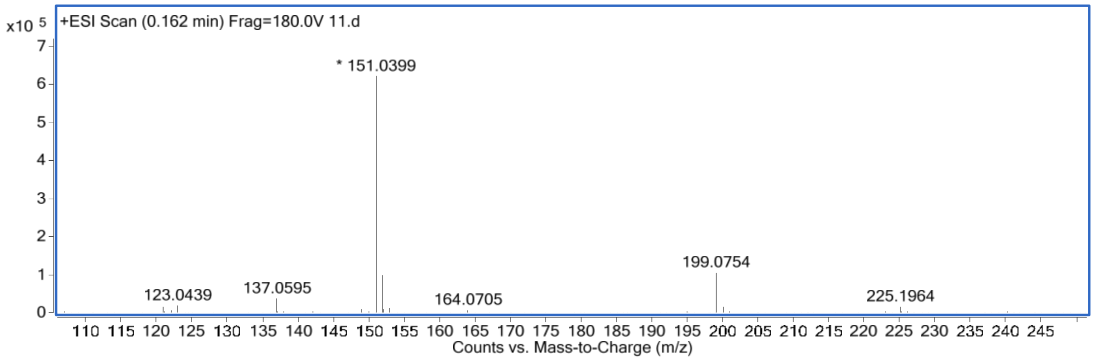

Supplement: Supplementary file 1 [file molecules-29-00831-s001.zip › molecules-2837896-supplementary.pdf]
